# Efficacy and Safety of Vernakalant for Cardioversion of Atrial Fibrillation and Flutter: A Systematic Review and Meta-Analysis

**DOI:** 10.3390/jcm15197406

**Published:** 2026-09-23

**Authors:** Abdulrahman A. Sayed, Leena Omair, Rawan Saud Aldreweash, Raaina Mahevish, Ahmed Turki Badi Alzahrani, Jarah Almutairy, Mothana Y. AlJaber, Hashim Hussein Alawami, Shahad Alamoudi, Yara Abdullah Khamag, Wala Ali Salman Alhajjaj, Kenan Alaa Badran, Ayman M. A. Mohamed

**Affiliations:** 1 Department of Clinical Pharmacy, College of Pharmacy, Jazan University, Jazan 45142, Saudi Arabia; dr.abdulrahman987@gmail.com; 2 College of Medicine, AlMaarefa University, Al Diriyah, Riyadh 13713, Saudi Arabiashahadalamoudi05@gmail.com (S.A.); walaa23ali@gmail.com (W.A.S.A.); 3 College of Medicine, Majmaah University, Majmaah 11952, Saudi Arabia; 4 College of Medicine, Alfaisal University, Riyadh 11533, Saudi Arabia; raaina.mahewish@gmail.com (R.M.); halawami@alfaisal.edu (H.H.A.); yarakhamag@gmail.com (Y.A.K.); drkenanbadran@gmail.com (K.A.B.); 5 College of Medicine, Albaha University, Albaha 65799, Saudi Arabia; dr.ahmedalzhrani.2@gmail.com; 6 College of Medicine, King Faisal University, Al-Ahsa 36362, Saudi Arabia; mothanaaljb@gmail.com

**Keywords:** vernakalant, atrial fibrillation, atrial flutter, pharmacological cardioversion, sinus rhythm, safety, efficacy

## Abstract

**Background/Objectives**: Vernakalant is an atrial-selective antiarrhythmic used for rapid pharmacological cardioversion of recent-onset atrial fibrillation. This systematic review and meta-analysis evaluated the efficacy and safety of vernakalant compared with placebo, active antiarrhythmic therapy, and electrical cardioversion. **Methods**: PubMed, Embase, Google Scholar, and the Cochrane Central Register of Controlled Trials (CENTRAL) were searched from inception to August 2026. Randomized clinical trials, clinical trials, and comparative observational studies evaluating vernakalant in adults with atrial fibrillation or atrial flutter were included. Dichotomous outcomes were pooled as risk ratios (RRs) with 95% confidence intervals (CIs) using the Mantel–Haenszel random-effects model in RevMan 5.4. **Results**: Twenty controlled studies involving 4523 patients were included, of whom 2349 received vernakalant and 2174 received control therapy. Vernakalant significantly increased successful conversion to sinus rhythm compared with all controls (1234/2244 (55%) vs. 991/2147 (46.2%); RR = 1.63; 95% CI [1.34, 1.99]; *p* < 0.00001). The effect was strongest versus placebo (RR = 5.33; 95% CI [1.95, 14.60]; *p* = 0.001) and remained significant versus procainamide (111/178 (62.3%) vs. 83/172 (48.2%); RR = 1.29; 95% CI [1.07, 1.57]; *p* = 0.009) and flecainide (209/280 vs. 209/330 (63.3%); RR = 1.18; 95% CI [1.05, 1.33]; *p* = 0.007), but non-significant against amiodarone, propafenone, ibutilide, or electrical cardioversion. Safety outcomes did not differ significantly between groups, including all-cause mortality (RR = 1.23), drug discontinuation (RR = 0.98), serious adverse events (RR = 0.87), ventricular tachycardia (RR = 0.68), bradycardia (RR = 1.31), and hypotension (RR = 0.80). **Conclusions**: Vernakalant was associated with higher rates of conversion to sinus rhythm, while no statistically significant differences were observed in major safety outcomes. These findings support its use as a pharmacological option for rapid rhythm restoration in appropriately selected patients.

## 1. Introduction

Atrial fibrillation (AF) is the most common sustained cardiac arrhythmia and carries a substantial healthcare burden, including an increased risk of stroke, significant morbidity, and mortality [1]. For hemodynamically stable patients presenting to the emergency department (ED) with recent-onset AF, early rhythm control strategies are highly recommended to prevent irreversible atrial remodeling and alleviate acute symptoms. Pharmacological cardioversion has emerged as an attractive first-line therapeutic option in this setting, as it avoids the procedural sedation, fasting requirements, and resource-intensive logistics associated with electrical cardioversion [1,2]. Vernakalant, a novel atrial-selective antiarrhythmic agent, was developed specifically for this rapid cardioversion indication. It selectively blocks atrial potassium currents and exerts a rate-dependent block of fast sodium channels, effectively prolonging the atrial effective refractory period with minimal impact on ventricular repolarization [3].

Vernakalant has been shown to be effective at restoring sinus rhythm in patients with recent-onset AF [4]. More recently, the RAFF4 randomized trial provided contemporary head-to-head evidence against procainamide, reporting higher conversion success with vernakalant (62.4% vs. 48.3%), a shorter time to conversion (21.8 vs. 44.7 min), and fewer attempted electrical cardioversions (33.7% vs. 44.2%), while adverse events were generally similar between groups [5]. Nevertheless, uncertainty regarding the safety profile of vernakalant has persisted, particularly because of concerns regarding adverse events and rare serious events. Previous evidence has identified bradycardia as a clinically relevant adverse event, while transient non-serious effects such as dysgeusia, paresthesia, and sneezing have also been reported [6]. These remaining uncertainties regarding safety across different patient populations further support the need for an updated synthesis of the available evidence.

To resolve these critical ambiguities, we conducted a comprehensive systematic review and meta-analysis of randomized controlled trials and comparative observational studies. The primary objective is to evaluate the efficacy of intravenous vernakalant in achieving rapid conversion to sinus rhythm in adult patients with AF or atrial flutter, providing a definitive, stratified comparison against placebo, electrical cardioversion, and specific active antiarrhythmic therapies. The secondary objective is to rigorously assess the drug’s safety profile by quantifying the relative risks of all-cause mortality, serious adverse events, bradycardia, and drug discontinuation. By integrating the most recent high-quality trial data, this analysis aims to clarify vernakalant’s clinical utility, tolerability burden, and safety margin in acute rhythm management.

## 2. Methods

This systematic review and meta-analysis was conducted in accordance with the Preferred Reporting Items for Systematic Reviews and Meta-Analyses (PRISMA) guidelines (File S1). The protocol was registered prospectively in PROSPERO under registration number [CRD420261451507]. All stages of study screening, eligibility assessment, data extraction, and quality assessment were performed independently by two reviewers, with disagreements resolved through discussion or consultation with a third reviewer when required.

### 2.1. Search Strategy and Data Sources

A comprehensive literature search was performed using PubMed, Embase, Google Scholar, and the Cochrane Central Register of Controlled Trials (CENTRAL) from database inception to 22 August 2026. The search was restricted to studies published in the English language. The search identified 541 records, of which 145 duplicates were removed, leaving 396 records for title and abstract screening. Of these, 356 records were excluded, and 40 reports were sought for retrieval. Following full-text assessment, 17 reports were excluded, and 20 studies met the eligibility criteria and were included in the meta-analysis (Figure 1).

The search strategy included combinations of keywords and medical subject terms related to vernakalant, RSD1235, Brinavess, atrial fibrillation, atrial flutter, cardioversion, sinus rhythm, and comparative antiarrhythmic therapy. Two independent reviewers screened the retrieved records by title and abstract, followed by full-text review of potentially eligible articles. Reference lists of included studies and relevant review articles were also reviewed manually to identify additional eligible studies. Any discrepancy regarding study eligibility was resolved by consensus, and unresolved disagreements were settled by a third reviewer. Extracted data were entered into a shared online spreadsheet. Both baseline and study characteristics, as well as outcome-specific event data, were collected. The complete search strategy is provided in the Supplementary File.

### 2.2. Inclusion and Exclusion Criteria

Studies were eligible for inclusion if they evaluated vernakalant for pharmacological cardioversion in adult patients with atrial fibrillation or atrial flutter and reported comparative data against placebo, antiarrhythmic drugs, or electrical cardioversion. Randomized controlled trials, clinical trials, and comparative observational studies were considered eligible if they provided extractable data for at least one efficacy or safety outcome. Observational studies were included to complement controlled trial evidence with comparative real-world data, particularly for active treatment comparisons with limited randomized evidence. Eligibility was not based on the availability of adjusted or propensity-score-matched estimates.

The primary efficacy outcome was successful conversion to sinus rhythm. It was extracted according to the assessment time point defined in each individual study. Where multiple follow-up periods were reported for the same safety outcome, data from the longest available follow-up were extracted to maximize event capture and ensure a consistent approach to outcome selection. Differences in conversion assessment windows, safety definitions, and follow-up durations across studies were considered potential sources of clinical heterogeneity. Safety outcomes included all-cause mortality, serious adverse events, drug discontinuation, and other adverse events. Studies were excluded if they were single-arm reports without a comparator, reviews, editorials, case reports, conference abstracts without sufficient extractable data, duplicate publications, or studies that did not report relevant outcomes for vernakalant and a control group.

### 2.3. Statistical Analysis

Primary meta-analyses and forest plots were performed using Review Manager (RevMan), version 5.4. Dichotomous outcomes were pooled as risk ratios (RRs) with corresponding 95% confidence intervals (CIs) using the Mantel–Haenszel random-effects model, given the expected clinical and methodological variability across studies and control interventions. Absolute event rates were also reported alongside pooled relative effects. Statistical significance was defined as a two-sided *p* value < 0.05. Heterogeneity was assessed using the chi-square test and quantified using the I^2^ statistic, with I^2^ values of 0–50%, 50–75%, and >75% interpreted as low, moderate, and high heterogeneity, respectively.

Subgroup analyses were performed according to comparator type for all efficacy and safety outcomes. Study-type subgroup analyses comparing clinical trials and observational studies were also performed when both study designs contributed data; these analyses were not applicable to serious adverse events and drug discontinuation because all contributing studies were clinical trials. Tests for subgroup differences were reported where applicable. In multi-arm studies with a shared vernakalant group contributing to more than one comparator contrast, the shared intervention group was divided approximately equally across comparisons, including events and denominators, to avoid double-counting participants.

Additional analyses were performed using Comprehensive Meta-Analysis (CMA), version 4.0. Separate univariable random-effects meta-regressions based on the log risk ratio were conducted to examine comparator type and study type as potential moderators, where applicable. Meta-regression results were summarized using regression coefficients, 95% CIs, *p* values, and the R^2^ analog for explained between-study variance. For random-effects analyses where estimable, 95% prediction intervals (PIs) were calculated to describe the expected range of true effects in a future comparable study.

Sensitivity analyses were performed by excluding studies enrolling exclusively atrial flutter or mixed atrial fibrillation/atrial flutter populations to evaluate the robustness of findings in AF-only studies. Sequential leave-one-out analyses were performed for each safety outcome to assess whether exclusion of individual studies altered the statistical significance of the pooled estimate.

### 2.4. Quality Assessment, Publication Bias and Certainty of Evidence

The methodological quality of randomized and clinical trials was assessed using the revised Cochrane risk-of-bias tool for randomized trials (RoB 2.0). This tool evaluates five domains: bias arising from the randomization process, bias due to deviations from intended interventions, bias due to missing outcome data, bias in outcome measurement, and bias in selection of the reported result. Each study was judged as having low risk of bias, some concerns, high risk of bias, or no information, with an overall judgment assigned accordingly. Observational cohort studies were evaluated using the Newcastle-Ottawa Scale, which assesses study quality across three domains: selection of study groups, comparability of cohorts, and ascertainment of outcomes. The results of quality assessment are provided in the Supplementary Files (Supplementary Figures S1 and S2, Supplementary Table S1). Overall, most observational studies were of high quality, while the included trials were generally of moderate to high quality.

Publication bias and small-study effects were assessed visually using funnel plots (Supplementary Figure S3A–G) and formally using Egger’s regression test, with a two-sided *p* value < 0.05 indicating statistically significant funnel-plot asymmetry. Significant Egger’s test findings were interpreted as evidence of possible small-study effects rather than definitive evidence of publication bias (Supplementary Table S3).

The certainty of evidence for each outcome was assessed using the GRADE approach across the domains of risk of bias, inconsistency, indirectness, imprecision, and publication bias, and was categorized as high, moderate, low, or very low certainty. The overall certainty of evidence ranged from very low to moderate across the evaluated outcomes (Supplementary Table S2).

## 3. Results

A total of 20 unique controlled studies comprising 4523 patients were included, with 2349 patients receiving vernakalant and 2174 receiving control therapy. Of these, 12 were randomized controlled trials, one was a clinical trial, and seven were observational studies. Control interventions included placebo, amiodarone, ibutilide, procainamide, flecainide, propafenone, and electrical cardioversion. Most studies enrolled patients with atrial fibrillation, three included mixed atrial fibrillation/atrial flutter populations, and one enrolled patients with atrial flutter alone. The study selection process is presented in Figure 1.

### 3.1. Successful Conversion to Sinus Rhythm

Among clinical trials, vernakalant was associated with a significantly higher likelihood of successful conversion to sinus rhythm than control therapy (707/1558 [45.4%] vs. 219/1014 [21.6%]; RR = 3.19; 95% CI [1.97, 5.15]; *p* < 0.00001), although heterogeneity was high (I^2^ = 90%). Among observational studies, conversion was also higher with vernakalant (527/686 [76.8%] vs. 772/1133 [68.1%]; RR = 1.11; 95% CI [1.01, 1.22]; *p* = 0.04; I^2^ = 62%). Overall, vernakalant was associated with higher conversion rates (1234/2244 [55.0%] vs. 991/2147 [46.2%]; RR = 1.63; 95% CI [1.34, 1.99]; *p* < 0.00001; I^2^ = 91%; 95% PI: [0.80, 2.78]) (Figure 2). The difference between study-type subgroups was significant (*p* < 0.0001).

Comparator-specific analysis showed a greater conversion rate with vernakalant versus placebo (RR = 5.33; 95% CI [1.95, 14.60]), procainamide (RR = 1.29; 95% CI [1.07, 1.57]), and flecainide (RR = 1.18; 95% CI [1.05, 1.33]). Comparisons with amiodarone (RR = 2.76; 95% CI [0.84, 9.08]), propafenone (RR = 1.04; 95% CI [0.81, 1.34]), ibutilide (RR = 1.13; 95% CI [0.94, 1.37]), and electrical cardioversion (RR = 0.90; 95% CI [0.79, 1.03]) did not demonstrate statistically significant differences (Supplementary Figure S4A).

### 3.2. All-Cause Mortality

Among clinical trials, mortality did not differ significantly between vernakalant and control groups (11/1381 [0.8%] vs. 3/842 [0.4%]; RR = 1.35; 95% CI [0.47, 3.84]; *p* = 0.57; I^2^ = 0%). The observational subgroup included one study and similarly showed no statistically significant difference (0/68 vs. 1/107; RR = 0.52; 95% CI [0.02, 12.63]; *p* = 0.69). Overall mortality remained uncommon and did not differ significantly between groups (11/1449 [0.8%] vs. 4/949 [0.4%]; RR = 1.23; 95% CI [0.46, 3.33]; *p* = 0.68; I^2^ = 0%) (Figure 3). Comparator-specific analyses likewise demonstrated no statistically significant differences (Supplementary Figure S4B).

### 3.3. Drug Discontinuation

All studies contributing to the drug-discontinuation outcome were clinical trials; therefore, study-type subgroup analysis was not applicable. Drug discontinuation occurred in 271/1557 (17.4%) vernakalant-treated patients and 119/948 (12.6%) controls, with no statistically significant overall difference (RR = 0.98; 95% CI [0.51, 1.88]; *p* = 0.94; I^2^ = 33%; 95% PI: [0.23, 4.11]) (Figure 4). Comparator-specific analysis showed lower discontinuation with vernakalant versus procainamide (RR = 0.26; 95% CI [0.09, 0.76]), whereas comparisons with placebo, amiodarone, and ibutilide were not statistically significant (Supplementary Figure S4C).

### 3.4. Serious Adverse Events

All studies contributing to serious adverse events were clinical trials. Serious adverse events occurred in 132/1385 (9.5%) vernakalant-treated patients and 81/759 (10.7%) controls, with no statistically significant difference (RR = 0.87; 95% CI [0.67, 1.14]; *p* = 0.31; I^2^ = 0%) (Figure 5). Comparator-specific analyses against placebo and amiodarone likewise did not demonstrate statistically significant differences (Supplementary Figure S4D).

### 3.5. Any Ventricular Tachycardia

Among clinical trials, ventricular tachycardia occurred in 56/1609 (3.5%) vernakalant-treated patients and 59/1009 (5.8%) controls (RR = 0.61; 95% CI [0.33, 1.12]; *p* = 0.11; I^2^ = 40%). Among observational studies, the corresponding rates were 2/330 (0.6%) and 2/744 (0.3%), respectively (RR = 1.79; 95% CI [0.31, 10.25]; *p* = 0.52; I^2^ = 0%). Overall, no statistically significant difference was observed (58/1939 [3.0%] vs. 61/1753 [3.5%]; RR = 0.68; 95% CI [0.38, 1.21]; *p* = 0.19; I^2^ = 34%; 95% PI: [0.18, 2.61]) (Figure 6). Comparator-specific analyses were generally non-significant, although the ibutilide subgroup favored vernakalant (RR = 0.17; 95% CI [0.03, 0.94]) (Supplementary Figure S4E).

### 3.6. Bradycardia

Among clinical trials, bradycardia occurred more frequently with vernakalant (51/1496 [3.4%] vs. 10/866 [1.2%]; RR = 2.21; 95% CI [1.16, 4.20]; *p* = 0.02; I^2^ = 0%). Conversely, the observational subgroup did not demonstrate a statistically significant difference (13/611 [2.1%] vs. 50/1038 [4.8%]; RR = 0.57; 95% CI [0.21, 1.58]; *p* = 0.28; I^2^ = 47%). When both study types were pooled, no statistically significant overall difference was observed (64/2107 [3.0%] vs. 60/1904 [3.2%]; RR = 1.31; 95% CI [0.67, 2.57]; *p* = 0.42; I^2^ = 44%; 95% PI: [0.18, 9.33]) (Figure 7). The difference between study-type subgroups was significant (*p* = 0.03). Comparator-specific analysis showed a higher risk versus placebo (RR = 2.76; 95% CI [1.27, 6.00]), whereas comparisons with ibutilide, procainamide, flecainide, and amiodarone were not statistically significant (Supplementary Figure S4F).

### 3.7. Hypotension

Among clinical trials, hypotension occurred in 46/935 (4.9%) vernakalant-treated patients and 32/673 (4.8%) controls (RR = 1.00; 95% CI [0.45, 2.18]; *p* = 0.99; I^2^ = 48%). Among observational studies, the corresponding rates were 4/330 (1.2%) and 48/803 (6.0%) (RR = 0.47; 95% CI [0.06, 3.80]; *p* = 0.48; I^2^ = 67%). Overall, hypotension did not differ significantly between groups (50/1265 [4.0%] vs. 80/1476 [5.4%]; RR = 0.80; 95% CI [0.36, 1.76]; *p* = 0.57; I^2^ = 57%; 95% PI: [0.07, 9.15]) (Figure 8). Comparator-specific analysis showed lower hypotension with vernakalant versus procainamide (RR = 0.19; 95% CI [0.06, 0.66]) and amiodarone (RR = 0.12; 95% CI [0.02, 0.87]), whereas other comparator subgroups were not statistically significant (Supplementary Figure S4G).

### 3.8. Meta-Regression & Egger’s Test

In separate univariable meta-regressions for successful conversion, both comparator type (coefficient = −0.9245; 95% CI [−1.3207, −0.5283]; *p* < 0.001; R^2^ analog = 0.13) and study type (coefficient = 0.6601; 95% CI [0.3662, 0.9541]; *p* < 0.001; R^2^ analog = 0.19) were significantly associated with treatment effect, although substantial residual heterogeneity remained. For bradycardia, comparator type (coefficient = −1.5283; *p* = 0.0047) and study type (coefficient = 1.6838; *p* = 0.0003) were also significant moderators. No statistically significant moderator effects were identified for the remaining evaluated outcomes. Egger’s regression indicated significant funnel-plot asymmetry for successful conversion (*p* = 0.02), bradycardia (*p* < 0.01), and mortality (*p* = 0.03), whereas no significant asymmetry was detected for serious adverse events, drug discontinuation, hypotension, or ventricular tachycardia (Supplementary Table S3).

### 3.9. Sensitivity Analysis

Sensitivity analyses excluding studies enrolling atrial flutter or mixed AF/AFL populations showed that the efficacy finding remained significant in the AF-only population (999/1785 vs. 876/1813; RR = 1.54; 95% CI [1.26, 1.88]; *p* < 0.0001; I^2^ = 90%) (Supplementary Figure S5A). AF-only analyses remained non-significant for mortality (RR = 1.12; 95% CI [0.39, 3.19]; *p* = 0.83; I^2^ = 0%), drug discontinuation (RR = 0.75; 95% CI [0.41, 1.37]; *p* = 0.35; I^2^ = 27%), serious adverse events (RR = 0.84; 95% CI [0.59, 1.19]; *p* = 0.33; I^2^ = 9%), bradycardia (RR = 1.07; 95% CI [0.55, 2.05]; *p* = 0.85; I^2^ = 16%), and hypotension (RR = 0.53; 95% CI [0.22, 1.25]; *p* = 0.15; I^2^ = 55%) (Supplementary Figure S5B–D,F,G). For ventricular tachycardia, the AF-only analysis showed a lower event risk with vernakalant (RR = 0.46; 95% CI [0.24, 0.86]; *p* = 0.02; I^2^ = 9%) (Supplementary Figure S5E).

A leave-one-out analysis within the placebo conversion subgroup showed that removal of Torp-Pedersen 2011 [18] reduced heterogeneity from I^2^ = 91% to 59%, while the effect remained significant (RR = 6.84; 95% CI [3.38, 13.85]; *p* < 0.00001). Sequential leave-one-out analyses for serious adverse events, drug discontinuation, bradycardia, hypotension, ventricular tachycardia, and all-cause mortality did not change the statistical significance status of their respective overall pooled estimates (Table 1).

## 4. Discussion

The primary findings of this systematic review and meta-analysis demonstrate that vernakalant is a potent pharmacological option for rapid cardioversion, achieving superior conversion rates of recent-onset AF in adult patients compared to placebo and procainamide. Its clinical utility is defined by its atrial selectivity and rapid onset, offering a viable alternative to existing antiarrhythmic agents in hemodynamically stable patients [3,26]. Unlike older agents that require prolonged infusion times, vernakalant’s electrophysiological profile allows for efficient rhythm restoration in acute settings [27].

Our findings are broadly consistent with previous evidence supporting vernakalant as an effective pharmacological cardioversion strategy. The recent Cochrane network meta-analysis also identified vernakalant among effective pharmacological options for acute cardioversion, while highlighting uncertainty in the comparative evidence across different cardioversion strategies [28]. Our analysis extends this evidence by incorporating newer head-to-head data, particularly the RAFF4 trial, in which vernakalant achieved higher conversion success than procainamide (62.4% vs. 48.3%) and a shorter time to conversion (21.8 vs. 44.7 min) [5]. These findings strengthen the contemporary comparative evidence for vernakalant while emphasizing the importance of interpreting its efficacy according to the specific comparator.

Safety remains an important consideration when selecting vernakalant for acute cardioversion. Previous literature has highlighted bradycardia as a clinically relevant adverse event associated with vernakalant [6]. In our pooled analysis, however, bradycardia did not differ significantly between vernakalant and control interventions (RR = 1.31; 95% CI [0.67, 2.57]; *p* = 0.42), while significant bradycardia was uncommon in the SPECTRUM real-world experience [29]. These findings are reassuring but should be interpreted cautiously, and clinically relevant bradycardia and other cardiovascular adverse events remain important considerations during treatment.

An important clinical dilemma identified in the literature surrounds the high relative risk of non-serious adverse events induced by vernakalant, such as transient dysgeusia and paresthesia [6,30]. Our pooled analysis reveals that the relative risk of overall drug discontinuation due to adverse events did not differ significantly between vernakalant and control arms (RR = 0.98; 95% CI [0.51, 1.88]; *p* = 0.94). This suggests that while vernakalant induces frequent, mild sensory phenomena, these side effects are tolerable and rarely lead to treatment failure or premature termination. This conclusion is supported by recent trial data; in the RAFF4 open-label trial, procainamide infusions had to be completely stopped in 8.7% of patients due to severe hypotension, whereas vernakalant did not cause higher rates of treatment discontinuation [5]. In contrast, our aggregated safety data demonstrate that the relative risk of hypotension for vernakalant is not significantly elevated (RR = 0.80).

These findings have practical implications for rhythm management in the ED. In the SPECTRUM study, first-line intravenous vernakalant achieved successful cardioversion in 67.8% of ED-treated episodes, with a median time to conversion of 11.0 min and a median hospital stay of 7.4 h [29]. Similarly, RAFF4 demonstrated that fewer patients treated with vernakalant underwent attempted electrical cardioversion compared with those receiving procainamide (33.7% vs. 44.2%) [5]. Rapid pharmacological conversion may therefore reduce the subsequent requirement for electrical cardioversion and its associated procedural requirements in appropriately selected patients. Together, these findings support vernakalant as a practical option for rapid rhythm restoration in haemodynamically stable patients with recent-onset AF in the ED. Patient-level characteristics may also influence the need and timing of active cardioversion. Mariani et al. identified previous spontaneous conversion, AF-related symptom duration <24 h, age ≥65 years, and female sex as predictors of spontaneous conversion to sinus rhythm during a 6 h observation period in hemodynamically stable patients presenting with symptomatic AF. These findings further support an individualized approach to rhythm-control decisions in the emergency setting [31].

These findings should be interpreted within current guideline-based patient selection. The 2024 ESC guidelines provide a Class I, Level A recommendation for intravenous vernakalant when pharmacological cardioversion of recent-onset AF is desired, excluding patients with recent acute coronary syndrome, heart failure with reduced ejection fraction, or severe aortic stenosis. Pharmacological cardioversion is intended for haemodynamically stable patients and, unlike electrical cardioversion, does not require fasting, sedation, or anaesthesia. For vernakalant specifically, the guidelines advise against its use in patients with arterial hypotension (systolic blood pressure < 100 mmHg), recent acute coronary syndrome within 1 month, NYHA class III or IV heart failure, QT prolongation, or severe aortic stenosis [32]. More generally, pharmacological cardioversion is not recommended in patients with sinus node dysfunction, atrioventricular conduction disturbances, or prolonged QTc (>500 ms) unless the risks of proarrhythmia and bradycardia have been considered. Eligibility for cardioversion must also incorporate thromboembolic risk: early cardioversion is not recommended when AF duration exceeds 24 h without appropriate anticoagulation or transoesophageal echocardiographic exclusion of cardiac thrombus. Accordingly, our findings support intravenous vernakalant as a rapid pharmacological cardioversion option in appropriately selected, haemodynamically stable patients with recent-onset AF [32].

To facilitate the practical integration of these safety parameters and guideline recommendations into acute care, we propose a step-by-step clinical decision algorithm (Figure 9). This visual pathway is designed to assist emergency and cardiovascular practitioners in rapidly identifying optimal candidates for vernakalant, systematically ruling out high-risk structural and ischemic contraindications, and executing the standardized two-dose infusion protocol while minimizing adverse events.

The certainty of evidence varied across outcomes. Moderate-certainty evidence was available for serious adverse events and ventricular tachycardia in clinical trials, whereas low-certainty evidence supported the clinical-trial estimates for successful conversion to sinus rhythm, all-cause mortality, bradycardia, hypotension, and drug discontinuation. In contrast, evidence from observational studies was of very low certainty for successful conversion, all-cause mortality, ventricular tachycardia, bradycardia, and hypotension. Accordingly, findings from observational studies should be regarded as supportive and interpreted more cautiously than the clinical-trial evidence (Supplementary Table S2).

Despite the promising findings presented by this systematic review, several critical limitations must be accounted for. First, substantial statistical heterogeneity was observed in the primary efficacy analysis (I^2^ = 91%). Sensitivity analysis excluding observational studies showed that the treatment effect remained statistically significant when restricted to clinical trials (RR = 3.19; 95% CI [1.97, 5.15]; *p* < 0.00001), although heterogeneity remained high (I^2^ = 90%). Meta-regression further demonstrated that study type significantly moderated the treatment effect (*p* < 0.001), accounting for approximately 19% of the between-study variance. These findings suggest that differences between clinical trials and observational studies contributed to the observed heterogeneity but did not fully explain it, and the pooled findings should therefore be interpreted with appropriate consideration of study design. Observational studies provided complementary real-world evidence but remain inherently susceptible to selection bias and confounding by indication. Although several studies reported multivariable or propensity-score-adjusted estimates, such adjustment was not uniformly available and differed substantially across studies. Treatment selection in some cohorts was influenced by baseline clinical characteristics, contraindications, and physician preference; therefore, unmeasured residual confounding may have affected the magnitude of the pooled observational estimates. These findings support interpreting the observational evidence as complementary to, rather than equivalent to, the randomized evidence.

Second, all-cause mortality was an exceedingly rare event across the included cohorts. While the current aggregated data does not show a statistically significant increase in mortality (RR = 1.23; 95% CI [0.46, 3.33]), the absolute event rate remains too low to definitively rule out such events, highlighting the need for continued pharmacovigilance. Third, additional clinical heterogeneity arose from differences in patient populations, arrhythmia type, outcome definitions, and assessment periods across studies. Successful conversion was assessed at different study-defined time points, which may influence comparisons between rapidly and more slowly acting treatments. Similarly, safety outcomes were reported using varying definitions and follow-up durations; although we consistently extracted the longest available follow-up when multiple time points were reported, these differences may have affected pooled estimates. Evidence for atrial flutter was limited, with most included studies evaluating AF; however, sensitivity analyses excluding AFL-only and mixed AF/AFL populations generally supported the robustness of the principal findings. Finally, while funnel-plot asymmetry for successful conversion, bradycardia, and mortality may reflect small-study effects rather than definitive publication bias, the presence of publication bias for these outcomes cannot be entirely ruled out and remains a potential limitation.

To advance the precision integration of vernakalant into cardiovascular practice, future clinical research must target these unresolved areas. Large-scale prospective registries are required to explicitly track 30-day and 6-month recurrence rates of atrial fibrillation following successful vernakalant cardioversion. Dedicated individual patient data (IPD) meta-analyses should be conducted to establish robust phenotyping algorithms, identifying how clinical variables such as age, body weight, and left atrial volume modify conversion success. Finally, well-powered head-to-head randomized controlled trials directly comparing intravenous vernakalant to intravenous ibutilide are necessary, utilizing standardized severity grading networks to clarify relative proarrhythmic risks and precise tolerability burdens.

## 5. Conclusions

In summary, intravenous vernakalant was associated with higher conversion rates compared with placebo, procainamide, and flecainide. Comparisons with amiodarone, ibutilide, propafenone, and electrical cardioversion did not demonstrate statistically significant differences; however, these findings should not be interpreted as evidence of equivalence or non-inferiority. Similarly, no statistically significant increase was observed in major safety outcomes, although the available evidence does not exclude clinically important differences, particularly for rare events. Vernakalant therefore remains a pharmacological option for rapid rhythm restoration in appropriately selected, hemodynamically stable patients. Future well-powered head-to-head randomized trials are needed to better define its comparative efficacy and safety.

## Figures and Tables

**Figure 1:**
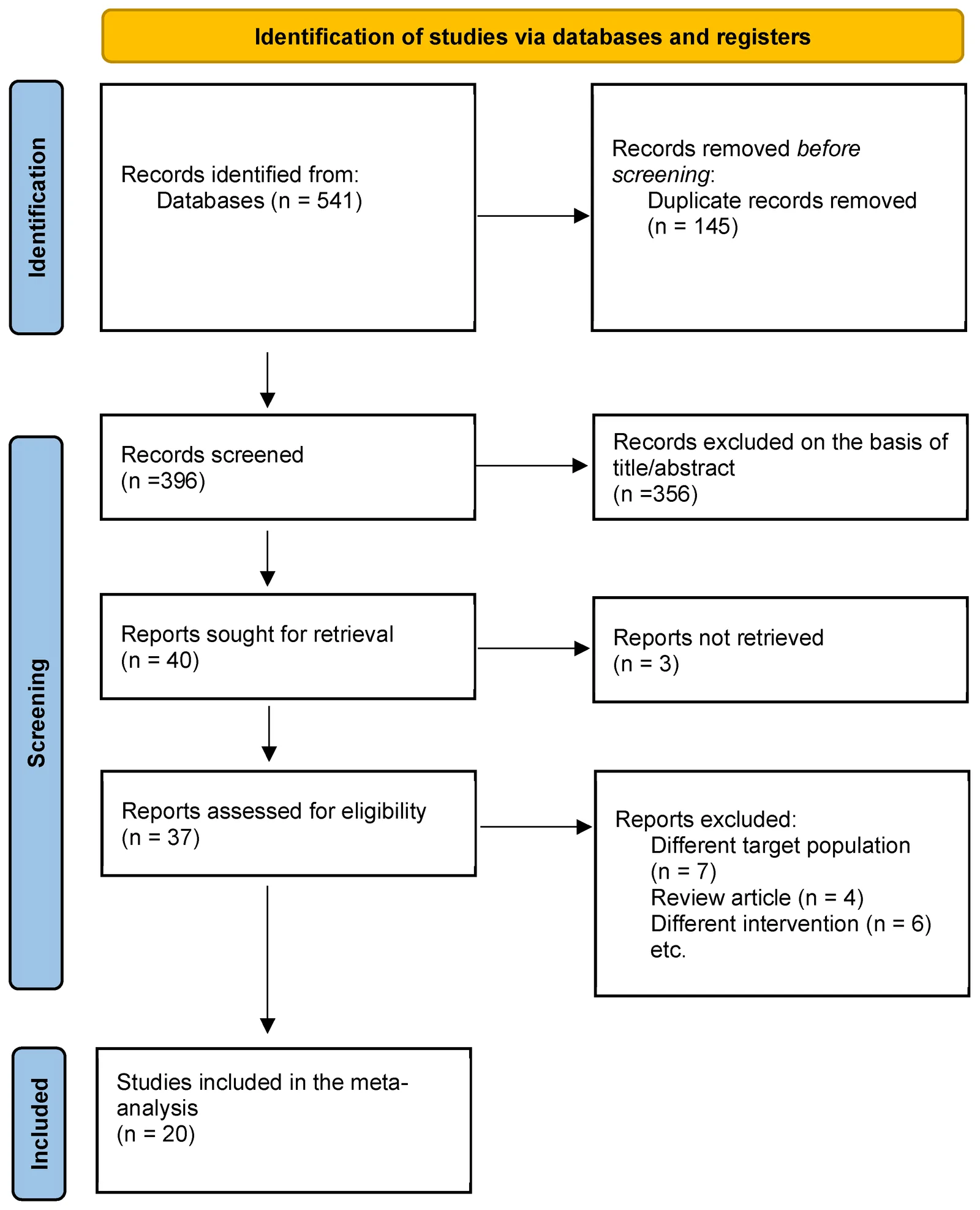
PRISMA flow diagram showing study identification, screening, eligibility assessment, and final inclusion.

**Figure 2:**
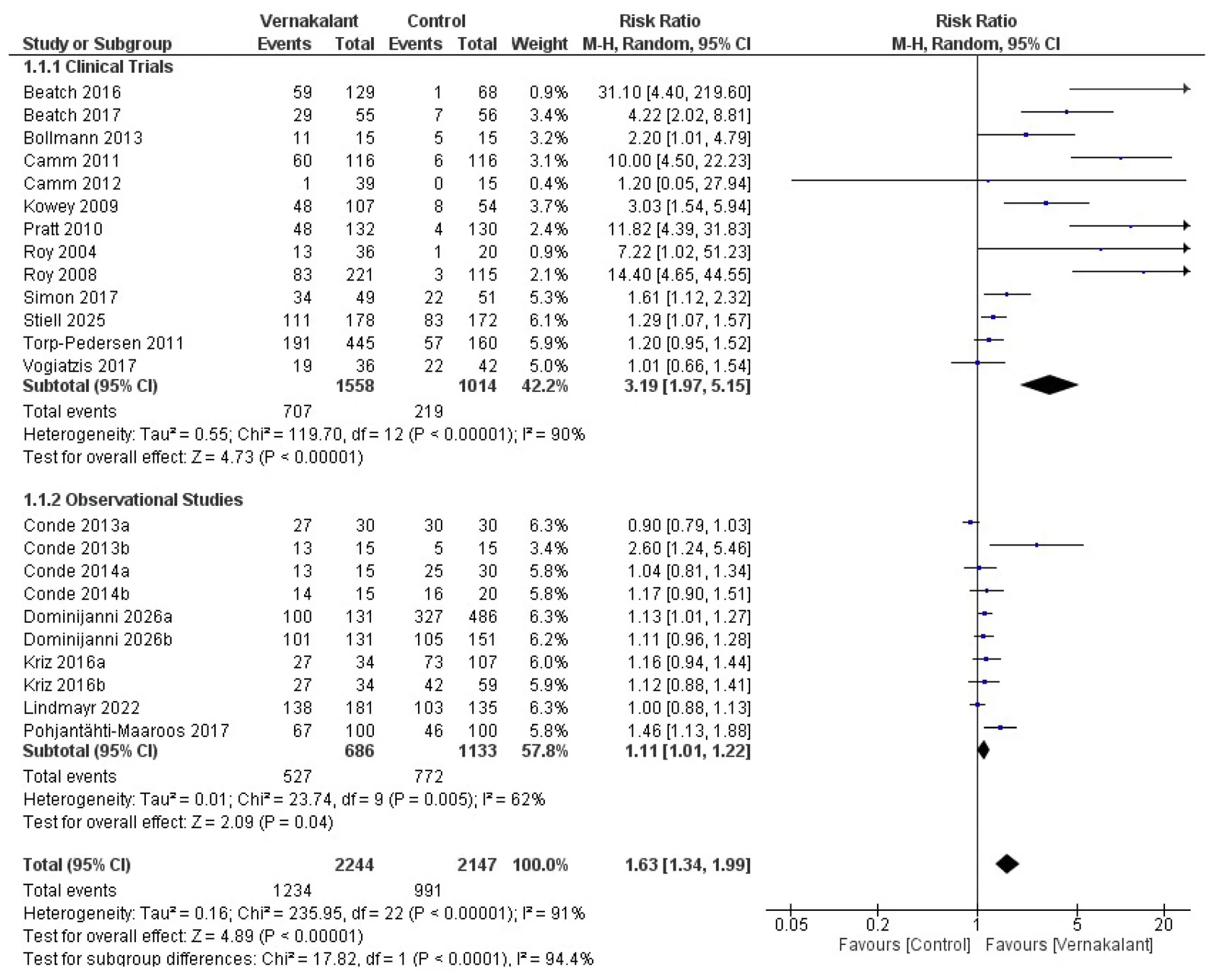
Forest plot comparing vernakalant versus the control interventions for successful conversion to sinus rhythm [5,7,8,9,10,11,12,13,14,15,16,17,18,19,20,21,22,23,24,25].

**Figure 3:**
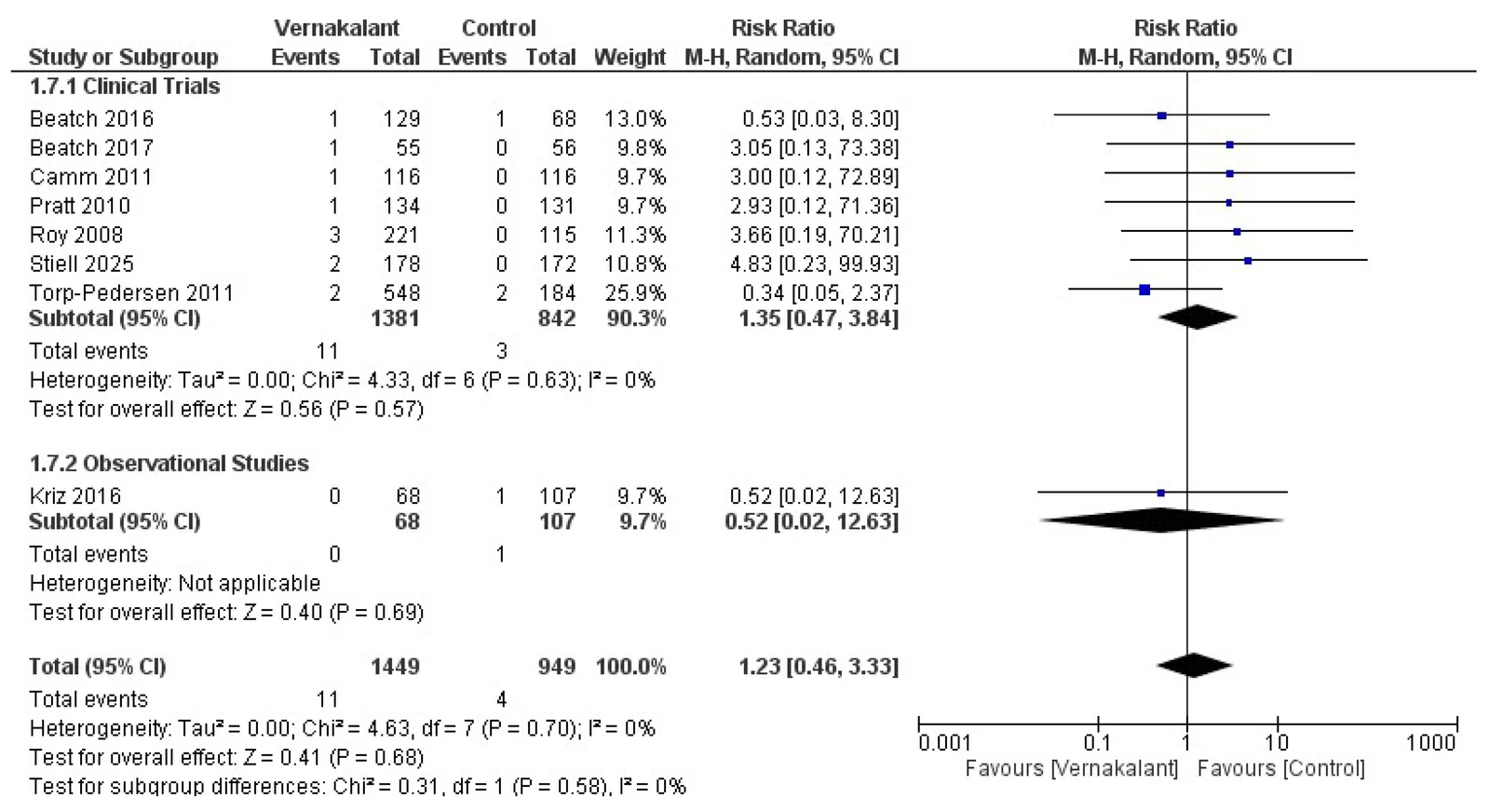
Forest plot comparing all-cause mortality between vernakalant and the control group [5,8,10,12,13,16,17,23].

**Figure 4:**
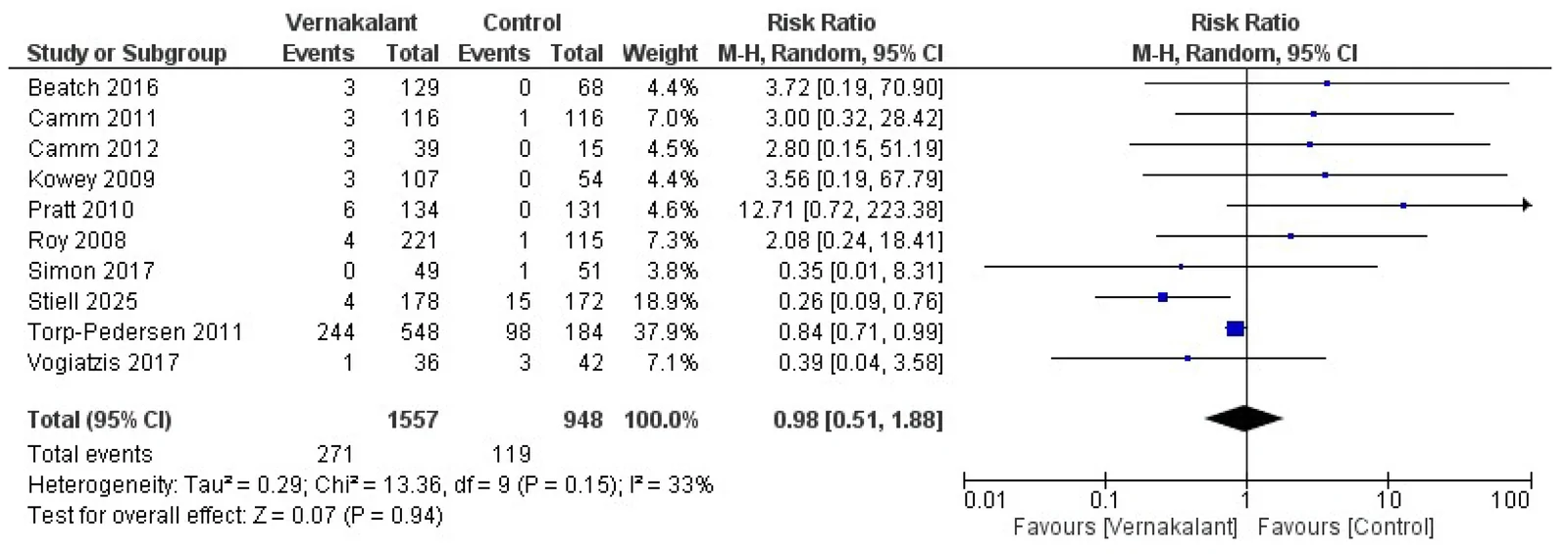
Forest plot comparing drug discontinuation between vernakalant and the control group [5,8,9,10,13,14,16,17,18,19].

**Figure 5:**
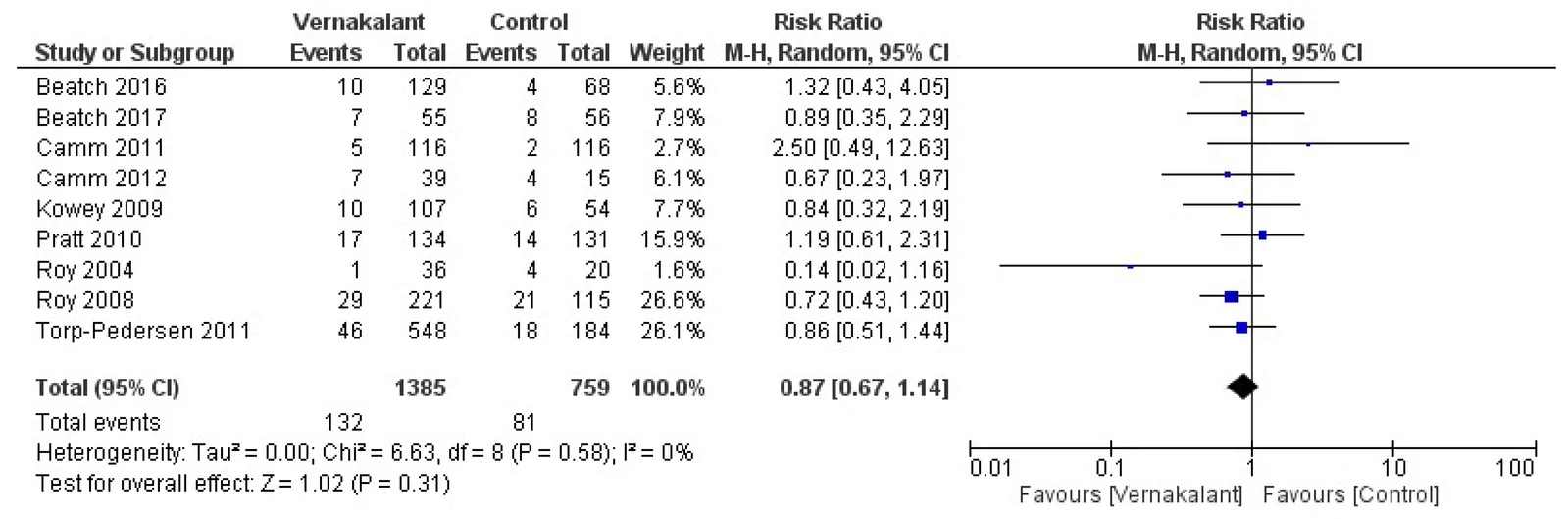
Forest plot comparing serious adverse events between vernakalant and the control group [8,9,10,12,13,14,15,16,18].

**Figure 6:**
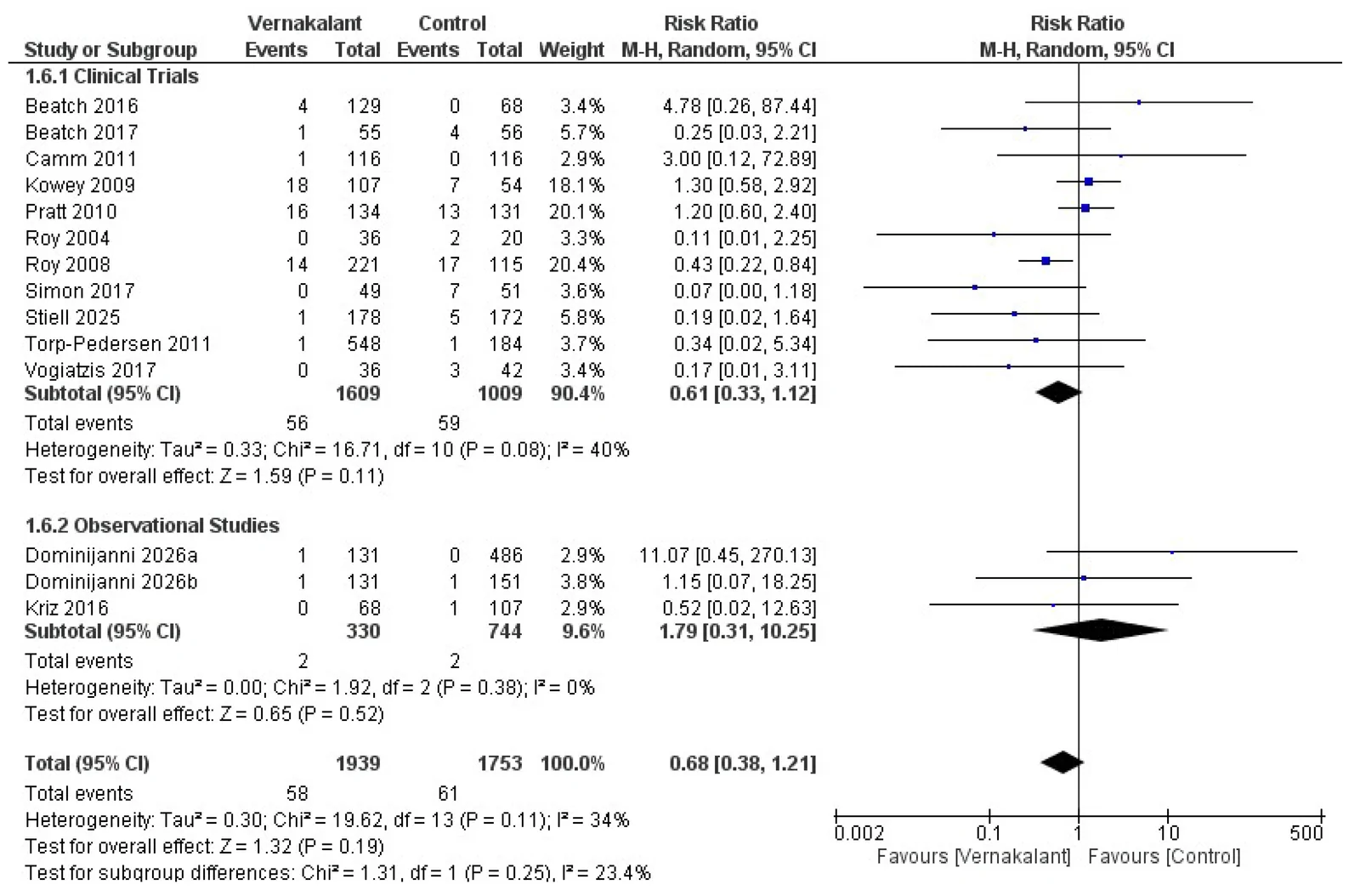
Forest plot comparing any ventricular tachycardia between vernakalant and the control group [5,8,9,10,12,13,15,16,17,18,19,22,23].

**Figure 7:**
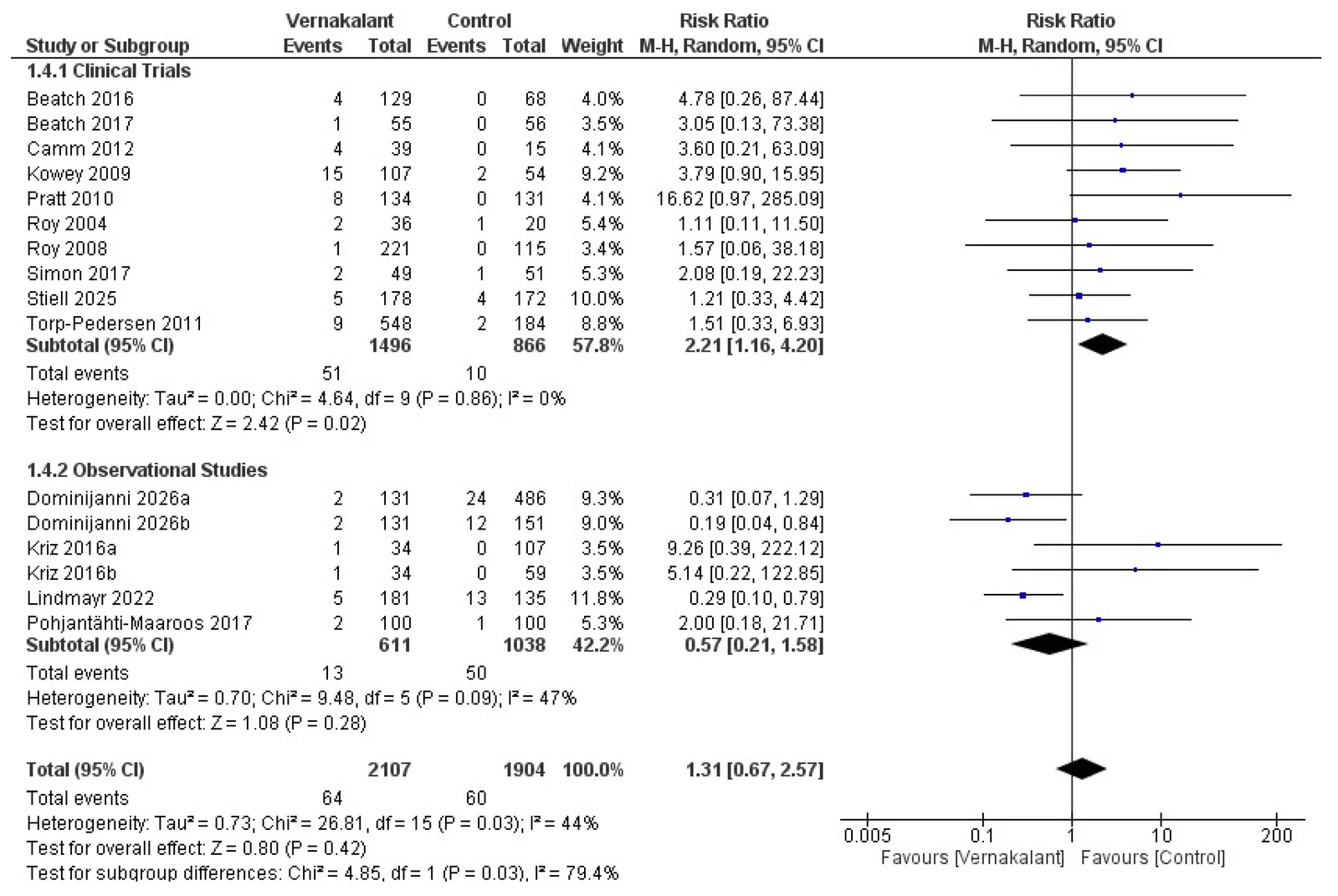
Forest plot comparing bradycardia between vernakalant and the control group [5,8,9,12,13,14,15,16,22,23,24,25].

**Figure 8:**
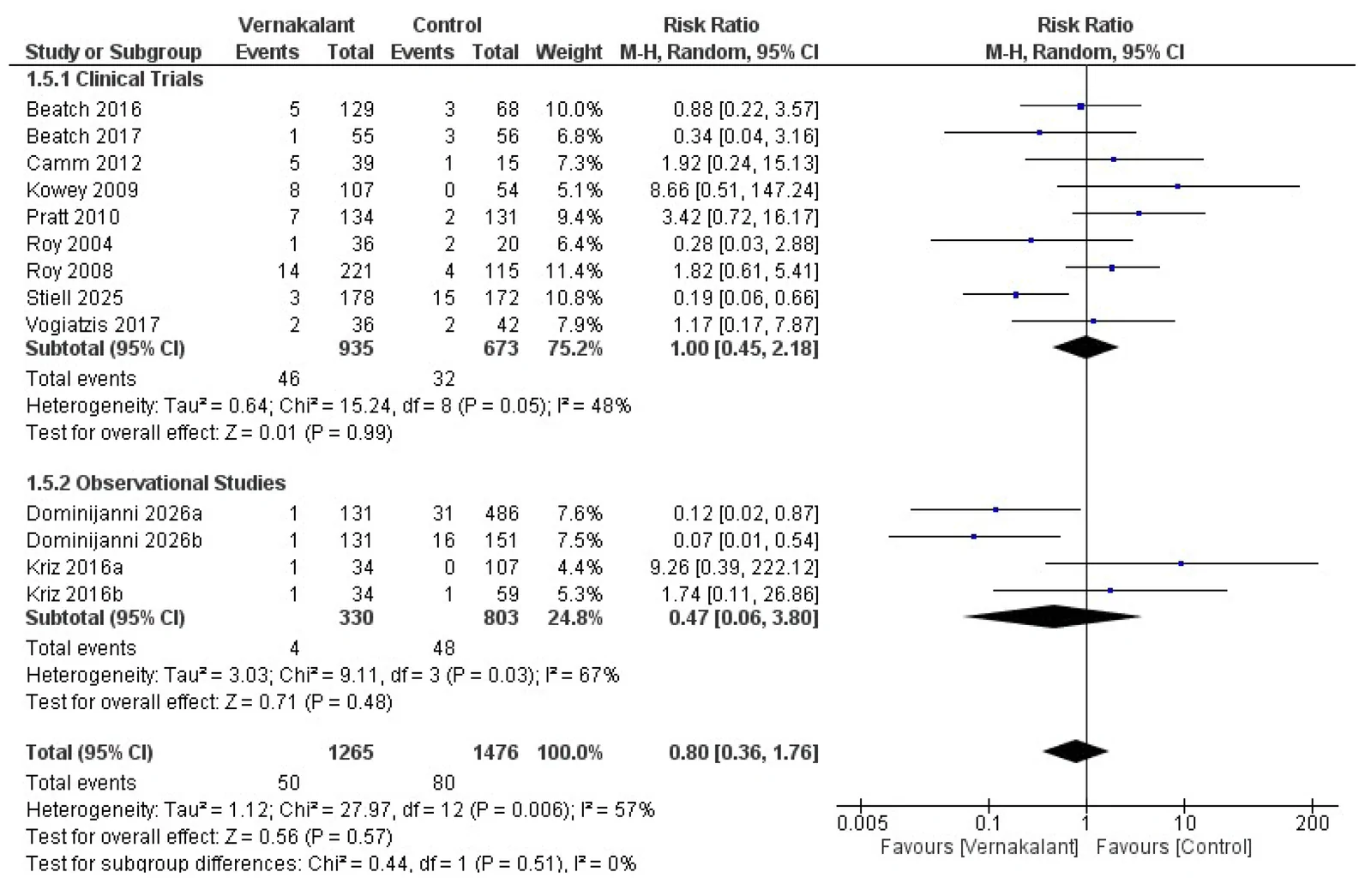
Forest plot comparing hypotension between vernakalant and the control group [5,8,9,12,13,14,15,16,19,22,23].

**Figure 9:**
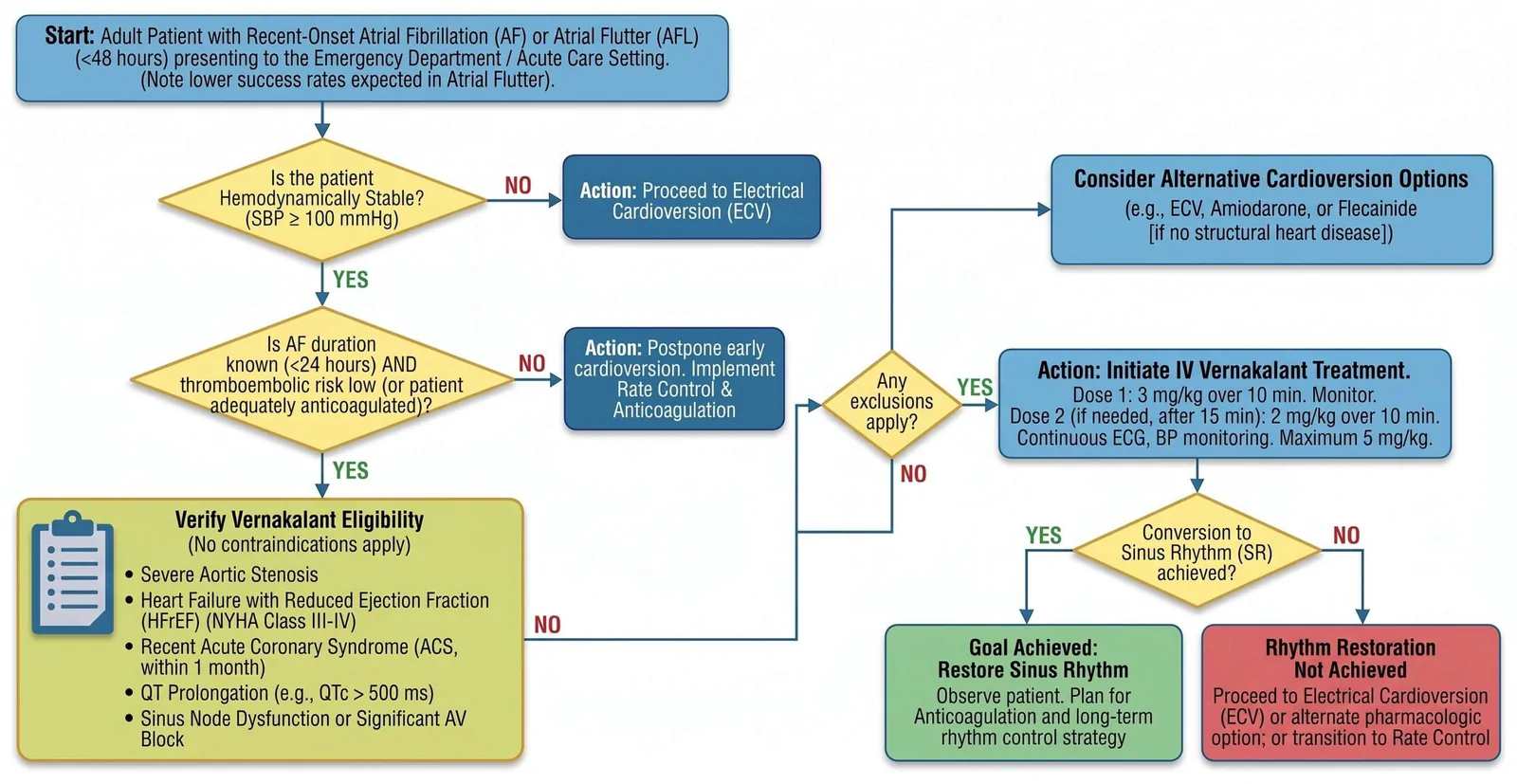
Proposed clinical algorithm for the selection and administration of intravenous vernakalant for rapid pharmacological cardioversion in emergency settings. Note: While vernakalant may be utilized for atrial flutter, it is primarily indicated for atrial fibrillation due to higher expected conversion rates. AF = Atrial Fibrillation; AFL = Atrial Flutter; ECV = Electrical Cardioversion; SBP = Systolic Blood Pressure; HFrEF = Heart Failure with reduced Ejection Fraction; ACS = Acute Coronary Syndrome.

**Table 1:** Study Characteristics of Included Vernakalant Studies.

Study ID	Intervention	Study Design	Location	Vernakalant (N)	Control (N)	Total Sample Size	Age, Mean (SD)	Gender (M/F)	Population	Trial Registration Number
Beatch 2016 [12]	Initial infusion 3 mg/kg, followed by a second infusion at 2 mg/kg.	Randomized Controlled Trial	Canada, Chile, Israel, Mexico, Peru, South Africa, USA	129	Placebo: 68	197	Vernakalant: 63.7 (12.7) Placebo: 60.8 (14.1)	121/76	Atrial Fibrillation	NCT00989001
Beatch 2017 [13]	Initial infusion 3 mg/kg, followed by a second infusion at 2 mg/kg.	Randomized Controlled Trial	Korea, Taiwan, India	55	Placebo: 56	111	Vernakalant: 60.7 (13.7) Placebo: 59.2 (12.0)	67/44	Atrial Fibrillation	NCT01174160.
Bollmann 2013 [11]	Vernakalant 3 mg/kg	Randomized Controlled Trial	Germany	15	Amiodarone: 15	30	N/A	N/A	Atrial Fibrillation	N/A
Camm 2011 [10]	Initial infusion at 3 mg/kg, followed by a second infusion at 2 mg/kg.	Randomized Controlled Trial	Australia, Canada, and Europe	116	Amiodarone: 116	232	Vernakalant: 63.1 (10.81) Amiodarone: 62.2 (11.63)	146/86	Atrial Fibrillation	NCT00668759
Kowey 2009 [9]	Initial infusion at 3 mg/kg, followed by a second infusion at 2 mg/kg.	Randomized Controlled Trial	Canada, the United States, Denmark, Italy, Poland, Argentina, India	107	Placebo: 54	161	Vernakalant: 68.3 (7.7) Placebo: 67.8 (6.4)	121/40	Atrial Fibrillation/Atrial Flutter	NCT00125320
Pratt 2010 [8]	Initial infusion at 3 mg/kg, followed by a second infusion at 2 mg/kg.	Randomized Controlled Trial	Canada, United States, Denmark, Sweden, Argentina, Mexico, Chile.	134	Placebo: 131	265	Vernakalant: 61.3 (14.5) Placebo: 61.7 (13.5)	178/84	Atrial Fibrillation/Atrial Flutter	NCT00115791
Roy 2004 [15]	0.5 mg/kg then 1.0 mg/kg OR 2.0 mg/kg then 3.0 mg/kg	Randomized Controlled Trial	United States, Canada	36	Placebo: 20	56	Vernakalant: 64.0 (range: 35–83) Placebo: 64.1 (range: 24–88)	34/22	Atrial Fibrillation	N/A
Roy 2008 [16]	Initial infusion at 3 mg/kg, followed by a second infusion at 2 mg/kg.	Randomized Controlled Trial	Canada, United States, Scandinavia	221	Placebo: 115	336	Vernakalant: 62.3 (13.7) Placebo: 61.5 (11.3)	234/102	Atrial Fibrillation	NCT00468767
Simon 2017 [17]	Initial infusion at 3 mg/kg, followed by a second infusion at 2 mg/kg.	Randomized Controlled Trial	Austria	49	Ibutilide: 51	100	Vernakalant: 56.2 (14.32) Ibutilide: 56.7 (15.77)	68/32	Atrial Fibrillation	NCT01447862
Torp-Pedersen 2011 [18]	150 mg, 300 mg, 500 mg	Randomized Controlled Trial	24 Countries	548	Placebo: 184	732	Vernakalant: 63.3 (10.6) Placebo: 62.8 (10.2)	492/240	Atrial Fibrillation	NCT00526136
Vogiatzis 2017 [19]	Initial infusion at 3 mg/kg, followed by a second infusion at 2 mg/kg.	Clinical Trial	Greece	36	Ibutilide: 42	78	Vernakalant: 62.44 (7.24) Ibutilide: 64.81 (6.10)	56/22	Atrial Fibrillation	N/A
Stiell 2025 [5]	Initial infusion at 3 mg/kg, followed by a second infusion at 2 mg/kg	Randomized Controlled Trial	Canada	178	Procainamide: 172	350	Vernakalant: 63.5 (15.0) Procainamide: 62.4 (15.2)	222/128	Atrial Fibrillation	NCT04485195
Lindmayr 2022 [24]	Initial infusion at 3 mg/kg, followed by a second infusion at 2 mg/kg	Prospective Observational Study	Austria	181	Ibutilide: 135	316	Vernakalant: 62 (IQR: 49–70); Ibutilide: 67 (IQR: 52–75)	195/121	Atrial Fibrillation/Atrial Flutter	N/A
Kriz 2016 [23]	Initial infusion at 3 mg/kg, followed by a second infusion at 2 mg/kg	Prospective Observational Study	Austria	68	Ibutilide: 107 Flecainide: 59 Amiodarone: 2	236	Vernakalant: 65 (15.3) Ibutilide: 69.2 (9.4) Flecainide: 64.8 (10.2)	103/133	Atrial Fibrillation	N/A
Pohjantähti-Maaroos 2017 [25]	Initial infusion at 3 mg/kg, followed by a second infusion at 2 mg/kg	Retrospective Observational Study	Finland	100	Flecainide: 100	200	Vernakalant: 59.3 (12.5) Flecainide: 55.3 (13.0)	131/69	Atrial Fibrillation	N/A
Camm 2012 [14]	Initial infusion at 3 mg/kg, followed by a second infusion at 2 mg/kg	Randomized Controlled Trial	Denmark, Canada, USA, Sweden	39	Placebo: 15	54	Vernakalant: 67 (11) Placebo: 69 (11)	38/16	Atrial Flutter	NCT00476112
Conde 2013a [7]	Initial infusion at 3 mg/kg, followed by a second infusion at 2 mg/kg	Observational Study	N/A	30	Electrical Cardioversion: 30	60	Overall Median Age: 65	42/18	Atrial Fibrillation	N/A
Conde 2013b [20]	Initial infusion at 3 mg/kg, followed by a second infusion at 2 mg/kg	Prospective Observational Study	N/A	15	Amiodarone: 15	30	N/A	N/A	Atrial Fibrillation	N/A
Conde 2014 [21]	Initial infusion at 3 mg/kg, followed by a second infusion at 2 mg/kg	Prospective Observational Study	Argentina	30	Propafenone: 30 Flecainide: 20	80	Vernakalant: 67 (56–69.5) Propafenone: 62 (54–66) Flecainide: 66 (55–69)	50/30	Atrial Fibrillation	N/A
Dominijanni 2026 [22]	I/V Vernakalant, median 3 mg/kg (IQR 3–5)	Retrospective Observational Study	Spain	262	Flecainide: 151 Amiodarone: 486	899	Vernakalant: 60.7 (12.7) Flecainide: 61.4 (12.5) Amiodarone: 67.9 (12.8)	468/431	Atrial Fibrillation	N/A

Notes: IQR; inter-quartile range.

## Data Availability

The original contributions presented in this study are included in the article and Supplementary Materials.

## Supplementary Materials

The following supporting information can be downloaded at https://www.mdpi.com/article/10.3390/jcm15197406/s1: Figure S1. Traffic-light plot showing study-level risk-of-bias judgments for included randomized and clinical trials using the revised Cochrane RoB 2.0 tool. Figure S2. Summary risk-of-bias plot showing the proportion of judgments across RoB 2.0 domains and overall risk of bias among included randomized and clinical trials. Figure S3. Funnel plots assessing publication bias across pooled outcomes. Figure S4A. Forest plot of successful conversion to sinus rhythm with vernakalant versus control, stratified by comparator type. Figure S4B. Forest plot of all-cause mortality with vernakalant versus control, stratified by comparator type. Figure S4C. Forest plot of drug discontinuation with vernakalant versus control, stratified by comparator type. Figure S4D. Forest plot of serious adverse events with vernakalant versus control, stratified by comparator type. Figure S4E. Forest plot of any ventricular tachycardia with vernakalant versus control, stratified by comparator type. Figure S4F. Forest plot of bradycardia with vernakalant versus control, stratified by comparator type. Figure S4G. Forest plot of hypotension with vernakalant versus control, stratified by comparator type. Figure S5A. Forest plot of successful conversion to sinus rhythm in the sensitivity analysis restricted to atrial fibrillation-only studies. Figure S5B. Forest plot of all-cause mortality in the sensitivity analysis restricted to atrial fibrillation-only studies. Figure S5C. Forest plot of drug discontinuation in the sensitivity analysis restricted to atrial fibrillation-only studies. Figure S5D. Forest plot of serious adverse events in the sensitivity analysis restricted to atrial fibrillation-only studies. Figure S5E. Forest plot of any ventricular tachycardia in the sensitivity analysis restricted to atrial fibrillation-only studies. Figure S5F. Forest plot of bradycardia in the sensitivity analysis restricted to atrial fibrillation-only studies. Figure S5G. Forest plot of hypotension in the sensitivity analysis restricted to atrial fibrillation-only studies. Table S1. Quality assessment of included cohort studies using the Newcastle–Ottawa Scale (NOS). Table S2. Summary of GRADE certainty of evidence for all evaluated outcomes. Table S3. Egger’s regression test for small-study effects across study outcomes. File S1. PRISMA 2020 Checklist.
