# Supplementary material for: Efficacy and Safety of Vernakalant for Cardioversion of Atrial Fibrillation and Flutter: A Systematic Review and Meta-Analysis
Source: J Clin Med. 2026 Sep 23;15(19):7406. doi: 10.3390/jcm15197406 (PMC13666120; doi:10.3390/jcm15197406)
Supplement: Supplementary file 1 [file jcm-15-07406-s001.zip › jcm-4492058-supplementary.pdf]

Supplementary Figure S1. Traffic-light plot showing study-level risk-of-bias judgments for included randomized and clinical trials using the revised Cochrane RoB 2.0 tool.

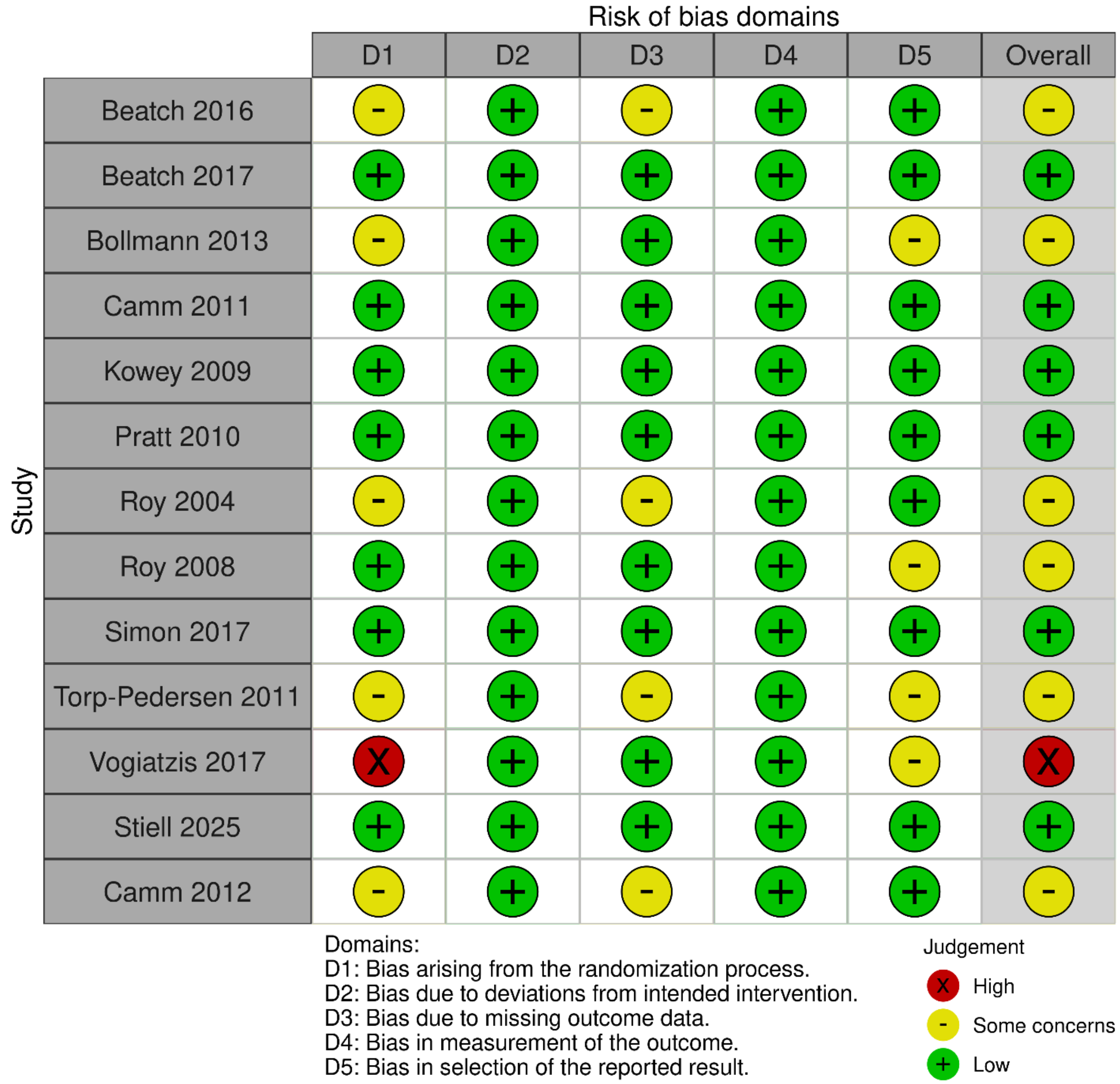

Supplementary Figure S2. Summary risk-of-bias plot showing the proportion of judgments across RoB 2.0 domains and overall risk of bias among included randomized and clinical trials

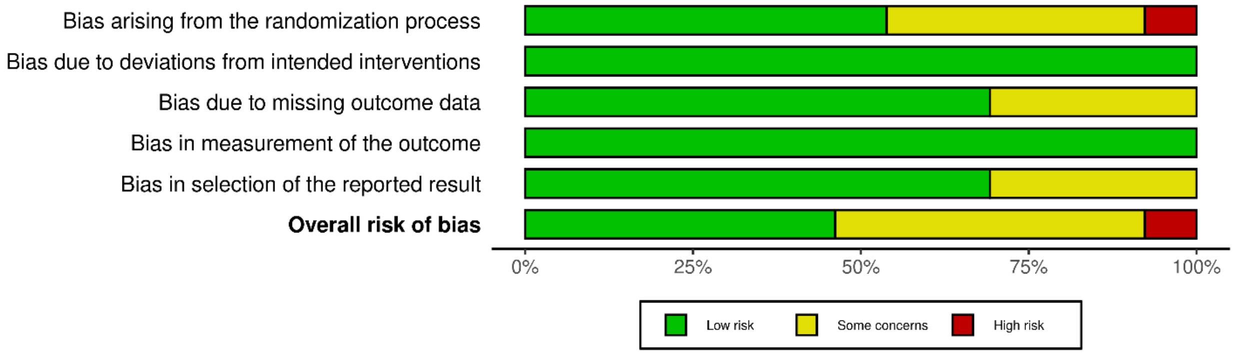

Supplementary Figure S3. Funnel plots assessing publication bias across pooled outcomes.

A. Funnel plot for successful conversion to sinus rhythm.

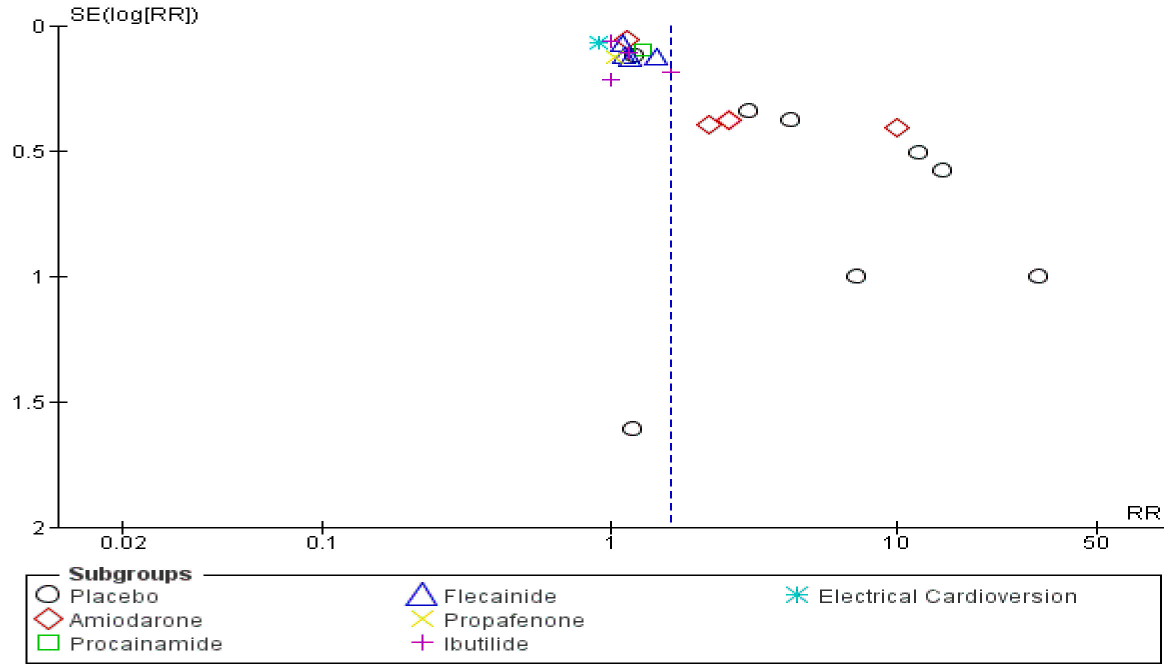

B. Funnel plot for all-cause mortality.

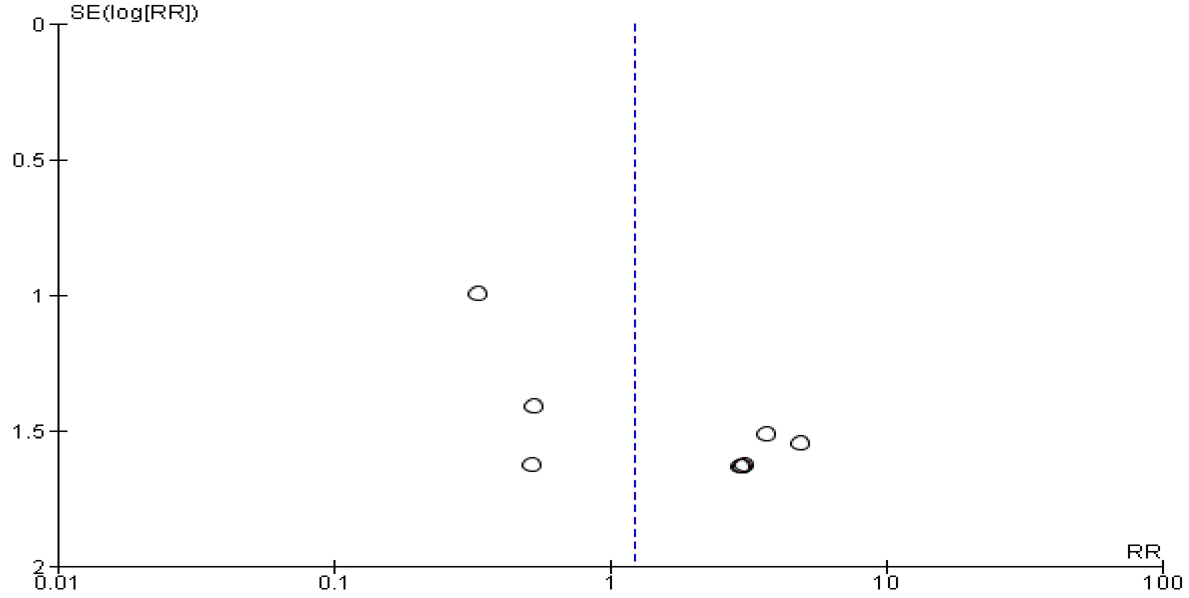

C. Funnel plot for drug discontinuation.

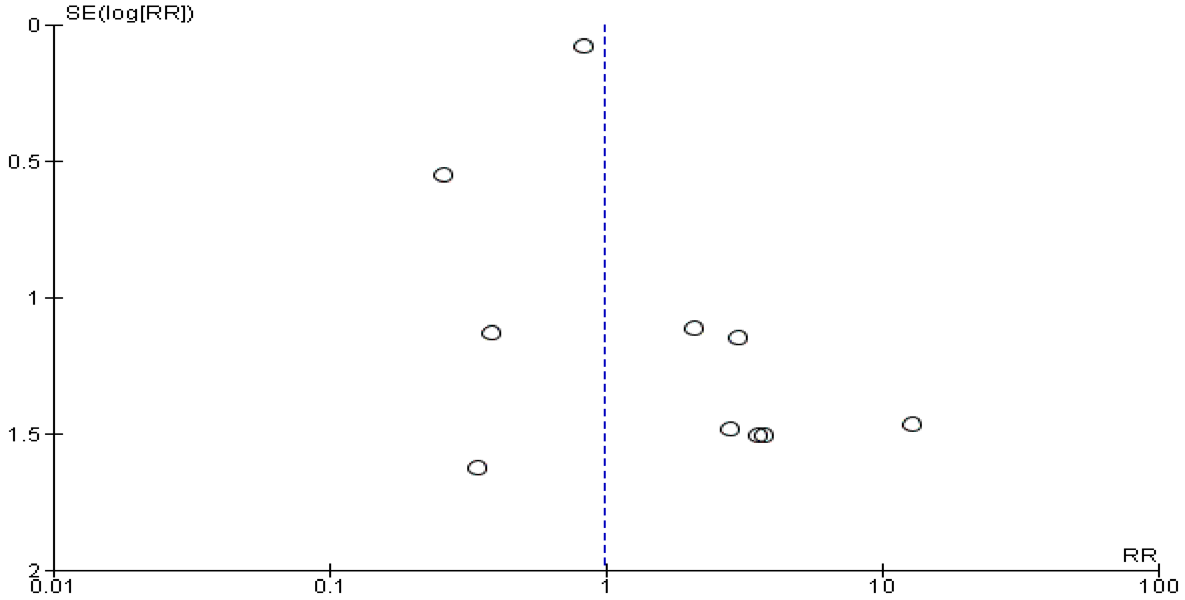

D. Funnel plot for serious adverse events.

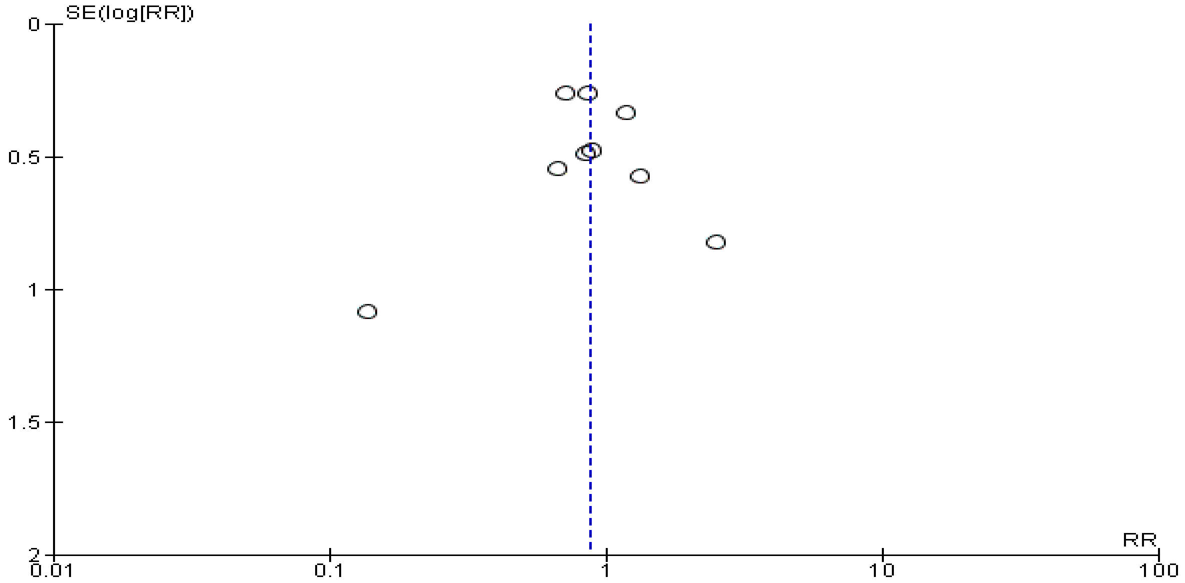

E. Funnel plot for any ventricular tachycardia.

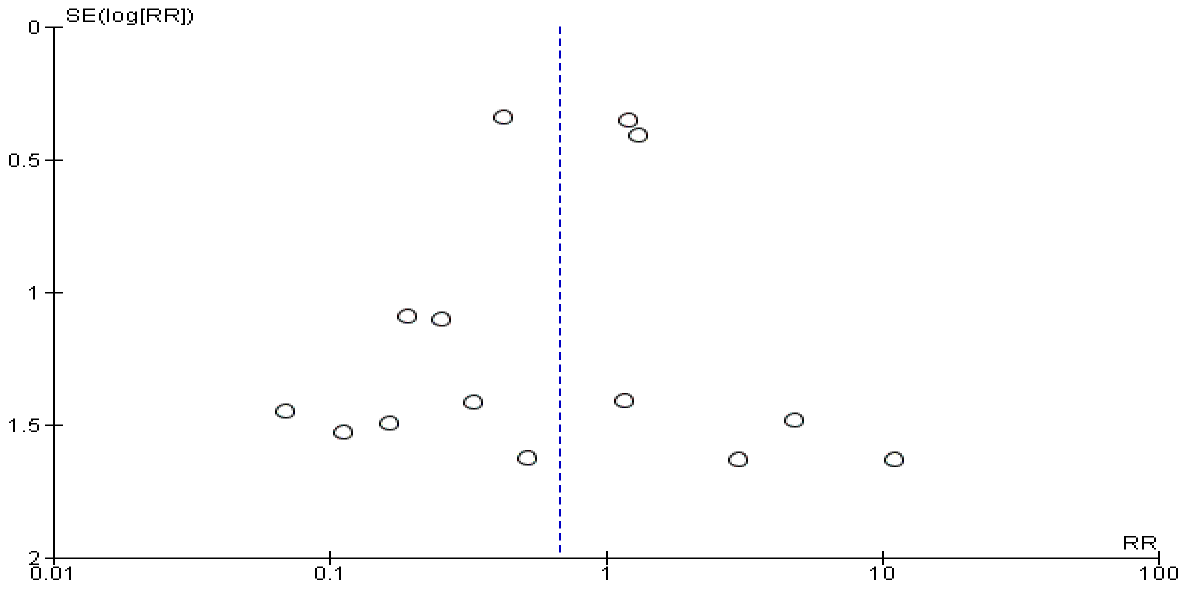

F. Funnel plot for bradycardia.

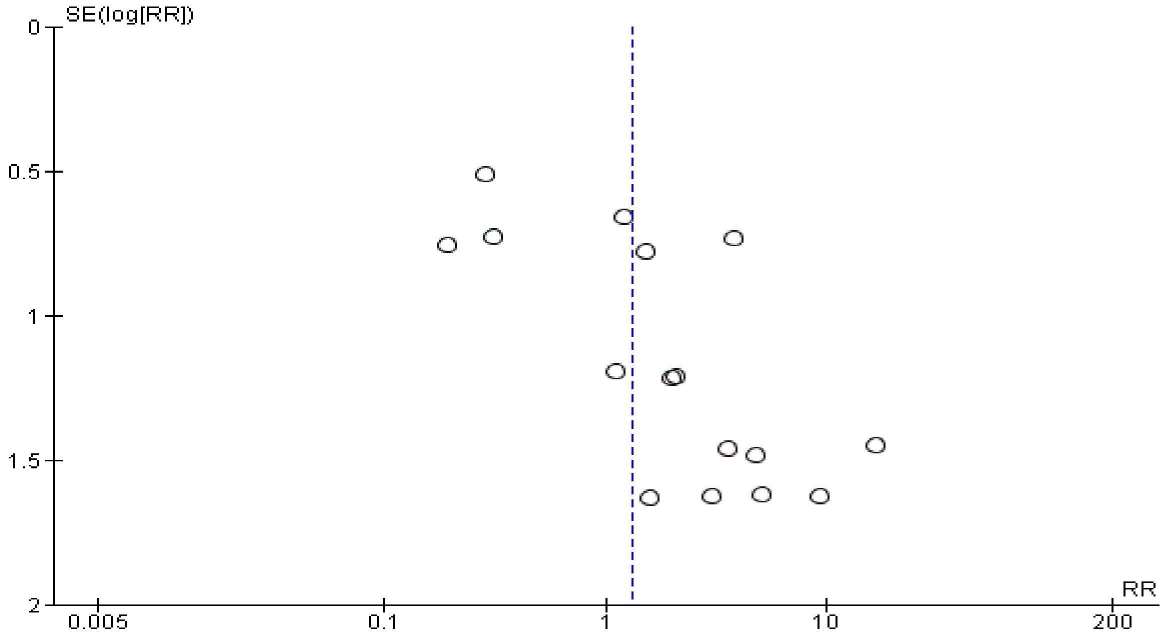

G. Funnel plot for hypotension.

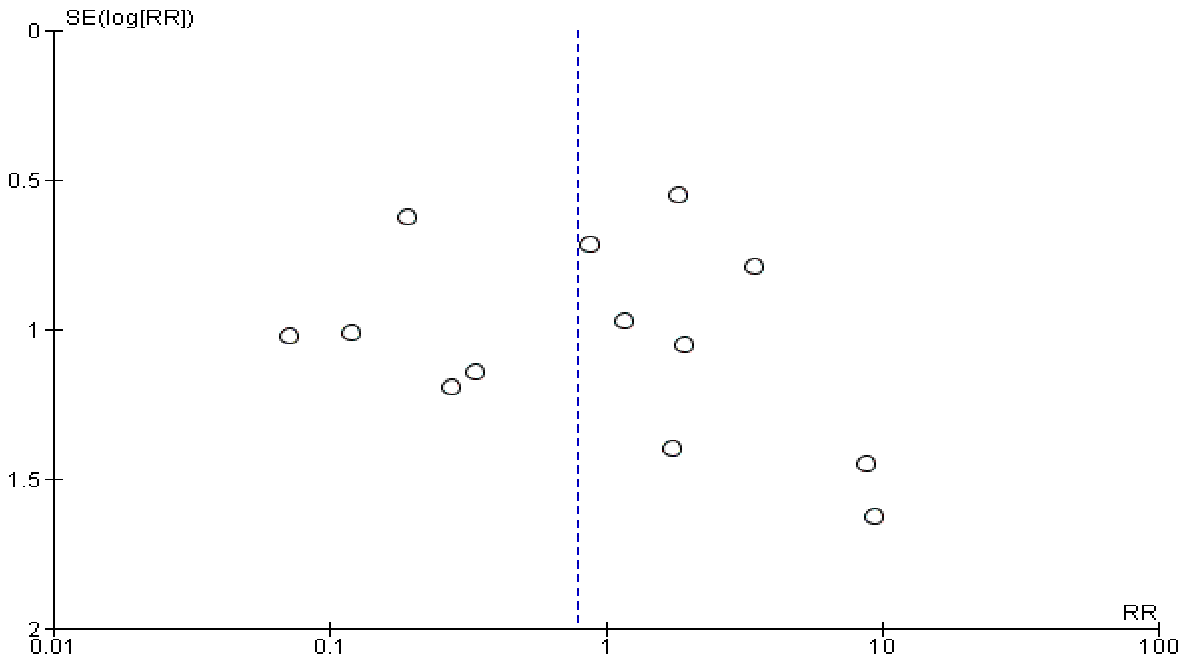

Supplementary Figure S4A. Forest plot of successful conversion to sinus rhythm with vernakalant versus control, stratified by comparator type.

Study or Subgroup	Vernakalant Events	Vernakalant Total	Control Events	Control Total	Weight	Risk Ratio M-H, Random, 95% CI
**1.1.1 Placebo**						
Beatch 2016	59	129	1	68	0.9%	31.10 [4.40, 219.60]
Beatch 2017	29	55	7	56	3.4%	4.22 [2.02, 8.81]
Camm 2012	1	39	0	15	0.4%	1.20 [0.05, 27.94]
Kowey 2009	48	107	8	54	3.7%	3.03 [1.54, 5.94]
Pratt 2010	48	132	4	130	2.4%	11.82 [4.39, 31.83]
Roy 2004	13	36	1	20	0.9%	7.22 [1.02, 51.23]
Roy 2008	83	221	3	115	2.1%	14.40 [4.65, 44.55]
Torp-Pedersen 2011	191	445	57	160	5.9%	1.20 [0.95, 1.52]
**Subtotal (95% CI)**		**1164**		**618**	**19.6%**	**5.33 [1.95, 14.60]**
Total events	472		81			
Heterogeneity: Tau² = 1.63; Chi² = 74.82, df = 7 (P < 0.00001); I² = 91%						
Test for overall effect: Z = 3.26 (P = 0.001)						
**1.1.2 Amiodarone**						
Bollmann 2013	11	15	5	15	3.2%	2.20 [1.01, 4.79]
Camm 2011	60	116	6	116	3.1%	10.00 [4.50, 22.23]
Conde 2013b	13	15	5	15	3.4%	2.60 [1.24, 5.46]
Dominijanni 2026a	100	131	327	486	6.3%	1.13 [1.01, 1.27]
**Subtotal (95% CI)**		**277**		**632**	**16.0%**	**2.76 [0.84, 9.08]**
Total events	184		343			
Heterogeneity: Tau² = 1.36; Chi² = 53.45, df = 3 (P < 0.00001); I² = 94%						
Test for overall effect: Z = 1.67 (P = 0.09)						
**1.1.3 Procainamide**						
Stiell 2025	111	178	83	172	6.1%	1.29 [1.07, 1.57]
**Subtotal (95% CI)**		**178**		**172**	**6.1%**	**1.29 [1.07, 1.57]**
Total events	111		83			
Heterogeneity: Not applicable						
Test for overall effect: Z = 2.61 (P = 0.009)						
**1.1.4 Flecainide**						
Conde 2014b	14	15	16	20	5.8%	1.17 [0.90, 1.51]
Dominijanni 2026b	101	131	105	151	6.2%	1.11 [0.96, 1.28]
Kriz 2016b	27	34	42	59	5.9%	1.12 [0.88, 1.41]
Pohjantähti-Maaroos 2017	67	100	46	100	5.8%	1.46 [1.13, 1.88]
**Subtotal (95% CI)**		**280**		**330**	**23.8%**	**1.18 [1.05, 1.33]**
Total events	209		209			
Heterogeneity: Tau² = 0.00; Chi² = 3.86, df = 3 (P = 0.28); I² = 22%						
Test for overall effect: Z = 2.69 (P = 0.007)						
**1.1.5 Propafenone**						
Conde 2014a	13	15	25	30	5.8%	1.04 [0.81, 1.34]
**Subtotal (95% CI)**		**15**		**30**	**5.8%**	**1.04 [0.81, 1.34]**
Total events	13		25			
Heterogeneity: Not applicable						
Test for overall effect: Z = 0.30 (P = 0.76)						
**1.1.6 Ibutilide**						
Kriz 2016a	27	34	73	107	6.0%	1.16 [0.94, 1.44]
Lindmayr 2022	138	181	103	135	6.3%	1.00 [0.88, 1.13]
Simon 2017	34	49	22	51	5.3%	1.61 [1.12, 2.32]
Vogiatzis 2017	19	36	22	42	5.0%	1.01 [0.66, 1.54]
**Subtotal (95% CI)**		**300**		**335**	**22.5%**	**1.13 [0.94, 1.37]**
Total events	218		220			
Heterogeneity: Tau² = 0.02; Chi² = 6.82, df = 3 (P = 0.08); I² = 56%						
Test for overall effect: Z = 1.32 (P = 0.19)						
**1.1.7 Electrical Cardioversion**						
Conde 2013a	27	30	30	30	6.3%	0.90 [0.79, 1.03]
**Subtotal (95% CI)**		**30**		**30**	**6.3%**	**0.90 [0.79, 1.03]**
Total events	27		30			
Heterogeneity: Not applicable						
Test for overall effect: Z = 1.52 (P = 0.13)						
**Total (95% CI)**		**2244**		**2147**	**100.0%**	**1.63 [1.34, 1.99]**
Total events	1234		991			
Heterogeneity: Tau² = 0.16; Chi² = 235.95, df = 22 (P < 0.00001); I² = 91%						
Test for overall effect: Z = 4.89 (P < 0.00001)						
Test for subgroup differences: Chi² = 24.53, df = 6 (P = 0.0004), I² = 75.5%						

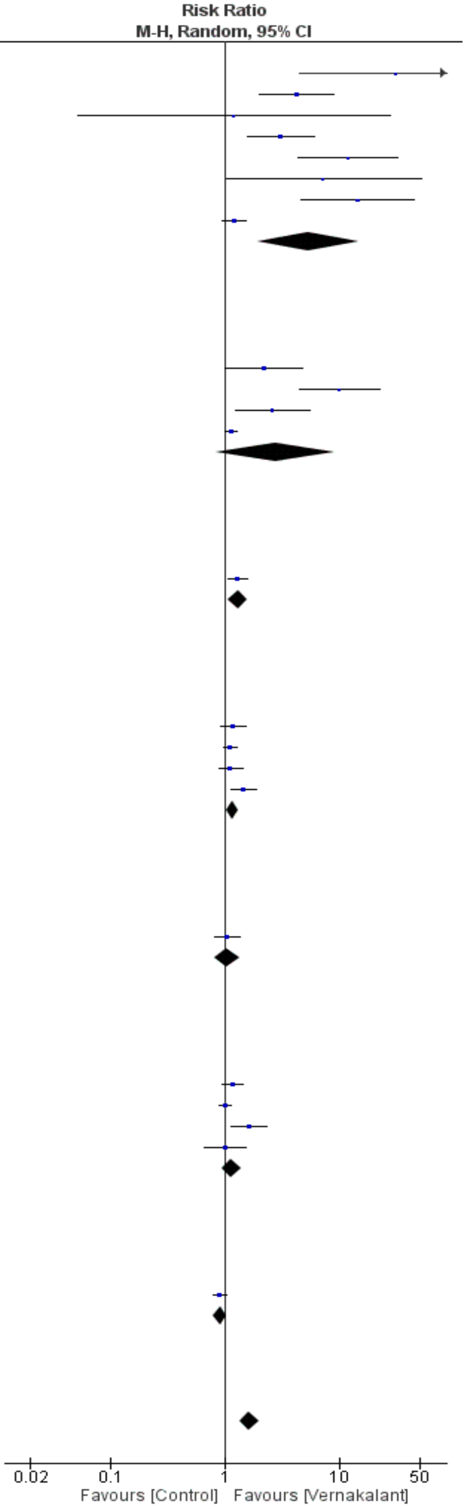

Supplementary Figure S4B. Forest plot of all-cause mortality with vernakalant versus control, stratified by comparator type.

	Vernakalant		Control			Risk Ratio
**Study or Subgroup**	**Events**	**Total**	**Events**	**Total**	**Weight**	**M-H, Random, 95% CI**
**1.7.1 Placebo**						
Beatch 2016	1	129	1	68	13.0%	0.53 [0.03, 8.30]
Beatch 2017	1	55	0	56	9.8%	3.05 [0.13, 73.38]
Pratt 2010	1	134	0	131	9.7%	2.93 [0.12, 71.36]
Roy 2008	3	221	0	115	11.3%	3.66 [0.19, 70.21]
Torp-Pedersen 2011	2	548	2	184	25.9%	0.34 [0.05, 2.37]
**Subtotal (95% CI)**		**1087**		**554**	**69.8%**	**0.99 [0.30, 3.26]**
Total events	8		3			
Heterogeneity: Tau² = 0.00; Chi² = 3.15, df = 4 (P = 0.53); I² = 0%						
Test for overall effect: Z = 0.01 (P = 0.99)						
**1.7.2 Amiodarone**						
Camm 2011	1	116	0	116	9.7%	3.00 [0.12, 72.89]
**Subtotal (95% CI)**		**116**		**116**	**9.7%**	**3.00 [0.12, 72.89]**
Total events	1		0			
Heterogeneity: Not applicable						
Test for overall effect: Z = 0.67 (P = 0.50)						
**1.7.3 Procainamide**						
Stiell 2025	2	178	0	172	10.8%	4.83 [0.23, 99.93]
**Subtotal (95% CI)**		**178**		**172**	**10.8%**	**4.83 [0.23, 99.93]**
Total events	2		0			
Heterogeneity: Not applicable						
Test for overall effect: Z = 1.02 (P = 0.31)						
**1.7.4 Ibutilide**						
Kriz 2016	0	68	1	107	9.7%	0.52 [0.02, 12.63]
**Subtotal (95% CI)**		**68**		**107**	**9.7%**	**0.52 [0.02, 12.63]**
Total events	0		1			
Heterogeneity: Not applicable						
Test for overall effect: Z = 0.40 (P = 0.69)						
**Total (95% CI)**		**1449**		**949**	**100.0%**	**1.23 [0.46, 3.33]**
Total events	11		4			
Heterogeneity: Tau² = 0.00; Chi² = 4.63, df = 7 (P = 0.70); I² = 0%						
Test for overall effect: Z = 0.41 (P = 0.68)						
Test for subgroup differences: Chi² = 1.49, df = 3 (P = 0.69), I² = 0%						

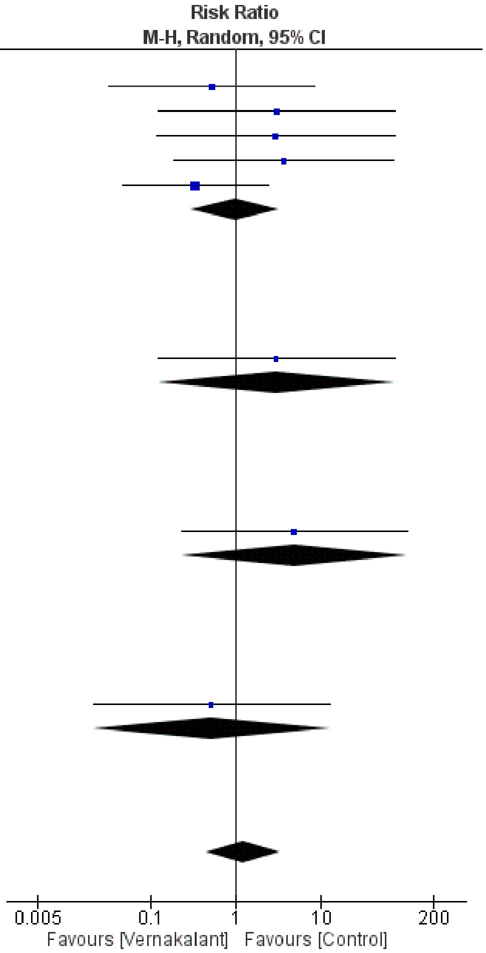

Supplementary Figure S4C. Forest plot of drug discontinuation with vernakalant versus control, stratified by comparator type.

	Vernakalant		Control			Risk Ratio
**Study or Subgroup**	**Events**	**Total**	**Events**	**Total**	**Weight**	**M-H, Random, 95% CI**
**1.3.1 Procainamide**						
Stiell 2025	4	178	15	172	18.9%	0.26 [0.09, 0.76]
**Subtotal (95% CI)**		**178**		**172**	**18.9%**	**0.26 [0.09, 0.76]**
Total events	4		15			
Heterogeneity: Not applicable						
Test for overall effect: Z = 2.45 (P = 0.01)						
**1.3.2 Placebo**						
Beatch 2016	3	129	0	68	4.4%	3.72 [0.19, 70.90]
Camm 2012	3	39	0	15	4.5%	2.80 [0.15, 51.19]
Kowey 2009	3	107	0	54	4.4%	3.56 [0.19, 67.79]
Pratt 2010	6	134	0	131	4.6%	12.71 [0.72, 223.38]
Roy 2008	4	221	1	115	7.3%	2.08 [0.24, 18.41]
Torp-Pedersen 2011	244	548	98	184	37.9%	0.84 [0.71, 0.99]
**Subtotal (95% CI)**		**1178**		**567**	**63.2%**	**1.73 [0.67, 4.46]**
Total events	263		99			
Heterogeneity: $Tau^2 = 0.45$; $Chi^2 = 7.31$, df = 5 (P = 0.20); $I^2 = 32\%$						
Test for overall effect: Z = 1.14 (P = 0.25)						
**1.3.3 Amiodarone**						
Camm 2011	3	116	1	116	7.0%	3.00 [0.32, 28.42]
**Subtotal (95% CI)**		**116**		**116**	**7.0%**	**3.00 [0.32, 28.42]**
Total events	3		1			
Heterogeneity: Not applicable						
Test for overall effect: Z = 0.96 (P = 0.34)						
**1.3.4 Ibutilide**						
Simon 2017	0	49	1	51	3.8%	0.35 [0.01, 8.31]
Vogiatzis 2017	1	36	3	42	7.1%	0.39 [0.04, 3.58]
**Subtotal (95% CI)**		**85**		**93**	**11.0%**	**0.37 [0.06, 2.31]**
Total events	1		4			
Heterogeneity: $Tau^2 = 0.00$; $Chi^2 = 0.00$, df = 1 (P = 0.95); $I^2 = 0\%$						
Test for overall effect: Z = 1.06 (P = 0.29)						
**Total (95% CI)**		**1557**		**948**	**100.0%**	**0.98 [0.51, 1.88]**
Total events	271		119			
Heterogeneity: $Tau^2 = 0.29$; $Chi^2 = 13.36$, df = 9 (P = 0.15); $I^2 = 33\%$						
Test for overall effect: Z = 0.07 (P = 0.94)						
Test for subgroup differences: $Chi^2 = 8.77$, df = 3 (P = 0.03), $I^2 = 65.8\%$						

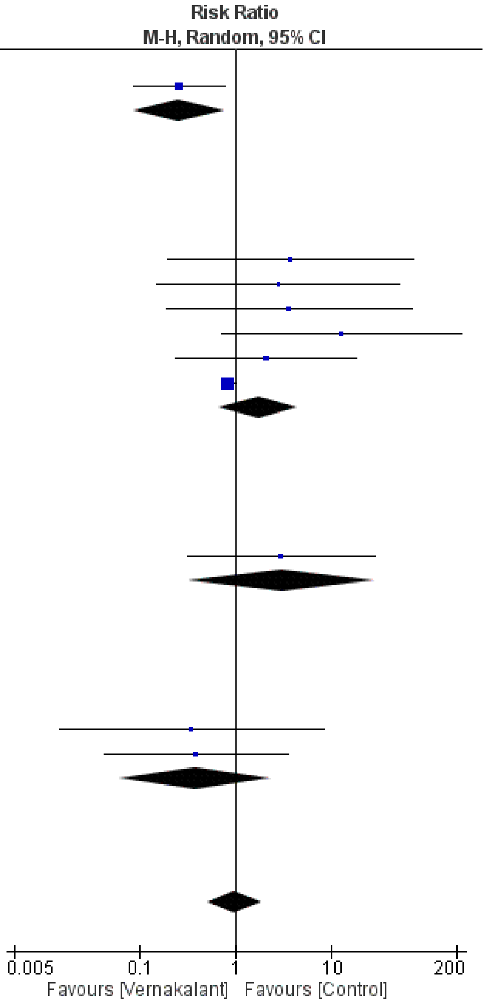

Supplementary Figure S4D. Forest plot of serious adverse events with vernakalant versus control, stratified by comparator type.

Study or Subgroup	Vernakalant Events	Vernakalant Total	Control Events	Control Total	Weight	Risk Ratio M-H, Random, 95% CI
**1.2.1 Placebo**						
Beatch 2016	10	129	4	68	5.6%	1.32 [0.43, 4.05]
Beatch 2017	7	55	8	56	7.9%	0.89 [0.35, 2.29]
Camm 2012	7	39	4	15	6.1%	0.67 [0.23, 1.97]
Kowey 2009	10	107	6	54	7.7%	0.84 [0.32, 2.19]
Pratt 2010	17	134	14	131	15.9%	1.19 [0.61, 2.31]
Roy 2004	1	36	4	20	1.6%	0.14 [0.02, 1.16]
Roy 2008	29	221	21	115	26.6%	0.72 [0.43, 1.20]
Torp-Pedersen 2011	46	548	18	184	26.1%	0.86 [0.51, 1.44]
**Subtotal (95% CI)**		**1269**		**643**	**97.3%**	**0.85 [0.65, 1.11]**
Total events	127		79			
Heterogeneity: Tau² = 0.00; Chi² = 4.96, df = 7 (P = 0.67); I² = 0%						
Test for overall effect: Z = 1.22 (P = 0.22)						
**1.2.2 Amiodarone**						
Camm 2011	5	116	2	116	2.7%	2.50 [0.49, 12.63]
**Subtotal (95% CI)**		**116**		**116**	**2.7%**	**2.50 [0.49, 12.63]**
Total events	5		2			
Heterogeneity: Not applicable						
Test for overall effect: Z = 1.11 (P = 0.27)						
**Total (95% CI)**		**1385**		**759**	**100.0%**	**0.87 [0.67, 1.14]**
Total events	132		81			
Heterogeneity: Tau² = 0.00; Chi² = 6.63, df = 8 (P = 0.58); I² = 0%						
Test for overall effect: Z = 1.02 (P = 0.31)						
Test for subgroup differences: Chi² = 1.67, df = 1 (P = 0.20), I² = 40.2%						

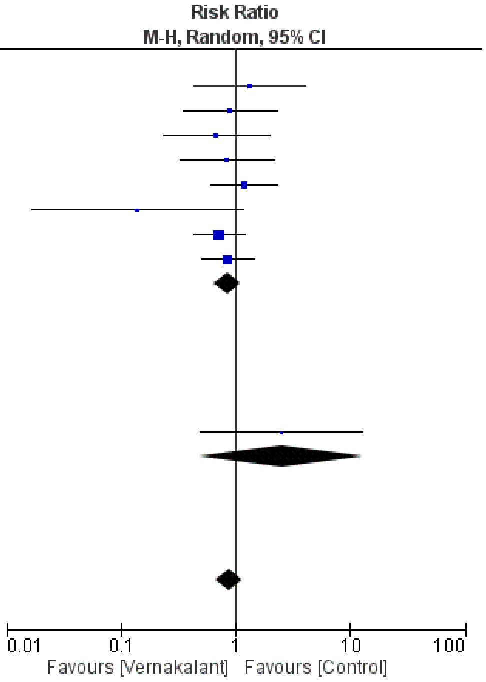

Supplementary Figure S4E. Forest plot of any ventricular tachycardia with vernakalant versus control, stratified by comparator type.

	Vernakalant		Control			Risk Ratio
Study or Subgroup	Events	Total	Events	Total	Weight	M-H, Random, 95% CI
**1.6.1 Placebo**						
Beatch 2016	4	129	0	68	3.4%	4.78 [0.26, 87.44]
Beatch 2017	1	55	4	56	5.7%	0.25 [0.03, 2.21]
Kowey 2009	18	107	7	54	18.1%	1.30 [0.58, 2.92]
Pratt 2010	16	134	13	131	20.1%	1.20 [0.60, 2.40]
Roy 2004	0	36	2	20	3.3%	0.11 [0.01, 2.25]
Roy 2008	14	221	17	115	20.4%	0.43 [0.22, 0.84]
Torp-Pedersen 2011	1	548	1	184	3.7%	0.34 [0.02, 5.34]
**Subtotal (95% CI)**		**1230**		**628**	**74.8%**	**0.75 [0.40, 1.41]**
Total events	54		44			
Heterogeneity: Tau² = 0.26; Chi² = 10.58, df = 6 (P = 0.10); I² = 43%						
Test for overall effect: Z = 0.90 (P = 0.37)						
**1.6.2 Amiodarone**						
Camm 2011	1	116	0	116	2.9%	3.00 [0.12, 72.89]
Dominijanni 2026a	1	131	0	486	2.9%	11.07 [0.45, 270.13]
**Subtotal (95% CI)**		**247**		**602**	**5.8%**	**5.76 [0.60, 55.03]**
Total events	2		0			
Heterogeneity: Tau² = 0.00; Chi² = 0.32, df = 1 (P = 0.57); I² = 0%						
Test for overall effect: Z = 1.52 (P = 0.13)						
**1.6.3 Ibutilide**						
Kriz 2016	0	68	1	107	2.9%	0.52 [0.02, 12.63]
Simon 2017	0	49	7	51	3.6%	0.07 [0.00, 1.18]
Vogiatzis 2017	0	36	3	42	3.4%	0.17 [0.01, 3.11]
**Subtotal (95% CI)**		**153**		**200**	**9.9%**	**0.17 [0.03, 0.94]**
Total events	0		11			
Heterogeneity: Tau² = 0.00; Chi² = 0.90, df = 2 (P = 0.64); I² = 0%						
Test for overall effect: Z = 2.04 (P = 0.04)						
**1.6.4 Procainamide**						
Stiell 2025	1	178	5	172	5.8%	0.19 [0.02, 1.64]
**Subtotal (95% CI)**		**178**		**172**	**5.8%**	**0.19 [0.02, 1.64]**
Total events	1		5			
Heterogeneity: Not applicable						
Test for overall effect: Z = 1.51 (P = 0.13)						
**1.6.5 Flecainide**						
Dominijanni 2026b	1	131	1	151	3.8%	1.15 [0.07, 18.25]
**Subtotal (95% CI)**		**131**		**151**	**3.8%**	**1.15 [0.07, 18.25]**
Total events	1		1			
Heterogeneity: Not applicable						
Test for overall effect: Z = 0.10 (P = 0.92)						
**Total (95% CI)**		**1939**		**1753**	**100.0%**	**0.68 [0.38, 1.21]**
Total events	58		61			
Heterogeneity: Tau² = 0.30; Chi² = 19.62, df = 13 (P = 0.11); I² = 34%						
Test for overall effect: Z = 1.32 (P = 0.19)						
Test for subgroup differences: Chi² = 7.54, df = 4 (P = 0.11), I² = 46.9%						

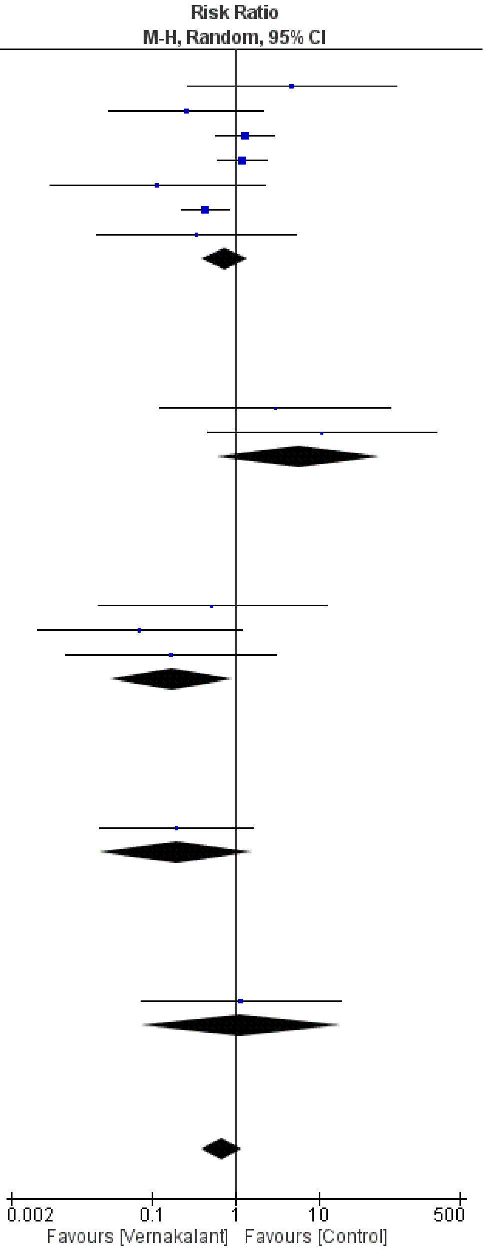

Supplementary Figure S4F. Forest plot of bradycardia with vernakalant versus control, stratified by comparator type.

Study or Subgroup	Vernakalant Events	Vernakalant Total	Control Events	Control Total	Weight	Risk Ratio M-H, Random, 95% CI
**1.4.1 Placebo**						
Beatch 2016	4	129	0	68	4.0%	4.78 [0.26, 87.44]
Beatch 2017	1	55	0	56	3.5%	3.05 [0.13, 73.38]
Camm 2012	4	39	0	15	4.1%	3.60 [0.21, 63.09]
Kowey 2009	15	107	2	54	9.2%	3.79 [0.90, 15.95]
Pratt 2010	8	134	0	131	4.1%	16.62 [0.97, 285.09]
Roy 2004	2	36	1	20	5.4%	1.11 [0.11, 11.50]
Roy 2008	1	221	0	115	3.4%	1.57 [0.06, 38.18]
Torp-Pedersen 2011	9	548	2	184	8.8%	1.51 [0.33, 6.93]
**Subtotal (95% CI)**		**1269**		**643**	**42.5%**	**2.76 [1.27, 6.00]**
Total events	44		5			
Heterogeneity: Tau² = 0.00; Chi² = 3.38, df = 7 (P = 0.85); I² = 0%						
Test for overall effect: Z = 2.56 (P = 0.01)						
**1.4.2 Ibutilide**						
Kriz 2016a	1	34	0	107	3.5%	9.26 [0.39, 222.12]
Lindmayr 2022	5	181	13	135	11.8%	0.29 [0.10, 0.79]
Simon 2017	2	49	1	51	5.3%	2.08 [0.19, 22.23]
**Subtotal (95% CI)**		**264**		**293**	**20.6%**	**1.18 [0.15, 9.38]**
Total events	8		14			
Heterogeneity: Tau² = 2.16; Chi² = 5.82, df = 2 (P = 0.05); I² = 66%						
Test for overall effect: Z = 0.16 (P = 0.87)						
**1.4.3 Procainamide**						
Stiell 2025	5	178	4	172	10.0%	1.21 [0.33, 4.42]
**Subtotal (95% CI)**		**178**		**172**	**10.0%**	**1.21 [0.33, 4.42]**
Total events	5		4			
Heterogeneity: Not applicable						
Test for overall effect: Z = 0.29 (P = 0.78)						
**1.4.4 Flecainide**						
Dominijanni 2026b	2	131	12	151	9.0%	0.19 [0.04, 0.84]
Kriz 2016b	1	34	0	59	3.5%	5.14 [0.22, 122.85]
Pohjantähti-Maaroos 2017	2	100	1	100	5.3%	2.00 [0.18, 21.71]
**Subtotal (95% CI)**		**265**		**310**	**17.7%**	**0.90 [0.11, 7.18]**
Total events	5		13			
Heterogeneity: Tau² = 1.99; Chi² = 4.99, df = 2 (P = 0.08); I² = 60%						
Test for overall effect: Z = 0.09 (P = 0.92)						
**1.4.5 Amiodarone**						
Dominijanni 2026a	2	131	24	486	9.3%	0.31 [0.07, 1.29]
**Subtotal (95% CI)**		**131**		**486**	**9.3%**	**0.31 [0.07, 1.29]**
Total events	2		24			
Heterogeneity: Not applicable						
Test for overall effect: Z = 1.61 (P = 0.11)						
**Total (95% CI)**		**2107**		**1904**	**100.0%**	**1.31 [0.67, 2.57]**
Total events	64		60			
Heterogeneity: Tau² = 0.73; Chi² = 26.81, df = 15 (P = 0.03); I² = 44%						
Test for overall effect: Z = 0.80 (P = 0.42)						
Test for subgroup differences: Chi² = 7.44, df = 4 (P = 0.11), I² = 46.2%						

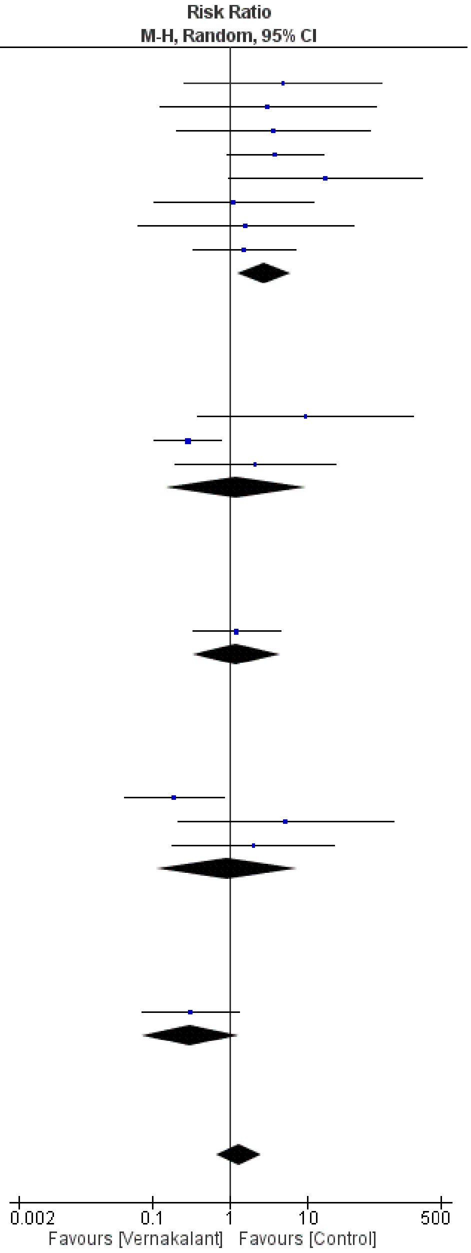

Supplementary Figure S4G. Forest plot of hypotension with vernakalant versus control, stratified by comparator type.

Study or Subgroup	Vernakalant Events	Vernakalant Total	Control Events	Control Total	Weight	Risk Ratio M-H, Random, 95% CI
**1.5.1 Placebo**						
Beatch 2016	5	129	3	68	10.0%	0.88 [0.22, 3.57]
Beatch 2017	1	55	3	56	6.8%	0.34 [0.04, 3.16]
Camm 2012	5	39	1	15	7.3%	1.92 [0.24, 15.13]
Kowey 2009	8	107	0	54	5.1%	8.66 [0.51, 147.24]
Pratt 2010	7	134	2	131	9.4%	3.42 [0.72, 16.17]
Roy 2004	1	36	2	20	6.4%	0.28 [0.03, 2.88]
Roy 2008	14	221	4	115	11.4%	1.82 [0.61, 5.41]
**Subtotal (95% CI)**		**721**		**459**	**56.4%**	**1.42 [0.70, 2.88]**
Total events	41		15			
Heterogeneity: Tau² = 0.14; Chi² = 7.08, df = 6 (P = 0.31); I² = 15%						
Test for overall effect: Z = 0.96 (P = 0.34)						
**1.5.2 Ibutilide**						
Kriz 2016a	1	34	0	107	4.4%	9.26 [0.39, 222.12]
Vogiatzis 2017	2	36	2	42	7.9%	1.17 [0.17, 7.87]
**Subtotal (95% CI)**		**70**		**149**	**12.3%**	**2.19 [0.34, 14.24]**
Total events	3		2			
Heterogeneity: Tau² = 0.36; Chi² = 1.20, df = 1 (P = 0.27); I² = 17%						
Test for overall effect: Z = 0.82 (P = 0.41)						
**1.5.3 Procainamide**						
Stiell 2025	3	178	15	172	10.8%	0.19 [0.06, 0.66]
**Subtotal (95% CI)**		**178**		**172**	**10.8%**	**0.19 [0.06, 0.66]**
Total events	3		15			
Heterogeneity: Not applicable						
Test for overall effect: Z = 2.64 (P = 0.008)						
**1.5.4 Amiodarone**						
Dominijanni 2026a	1	131	31	486	7.6%	0.12 [0.02, 0.87]
**Subtotal (95% CI)**		**131**		**486**	**7.6%**	**0.12 [0.02, 0.87]**
Total events	1		31			
Heterogeneity: Not applicable						
Test for overall effect: Z = 2.10 (P = 0.04)						
**1.5.5 Flecainide**						
Dominijanni 2026b	1	131	16	151	7.5%	0.07 [0.01, 0.54]
Kriz 2016b	1	34	1	59	5.3%	1.74 [0.11, 26.86]
**Subtotal (95% CI)**		**165**		**210**	**12.8%**	**0.31 [0.01, 7.60]**
Total events	2		17			
Heterogeneity: Tau² = 3.87; Chi² = 3.58, df = 1 (P = 0.06); I² = 72%						
Test for overall effect: Z = 0.72 (P = 0.47)						
**Total (95% CI)**		**1265**		**1476**	**100.0%**	**0.80 [0.36, 1.76]**
Total events	50		80			
Heterogeneity: Tau² = 1.12; Chi² = 27.97, df = 12 (P = 0.006); I² = 57%						
Test for overall effect: Z = 0.56 (P = 0.57)						
Test for subgroup differences: Chi² = 12.65, df = 4 (P = 0.01), I² = 68.4%						

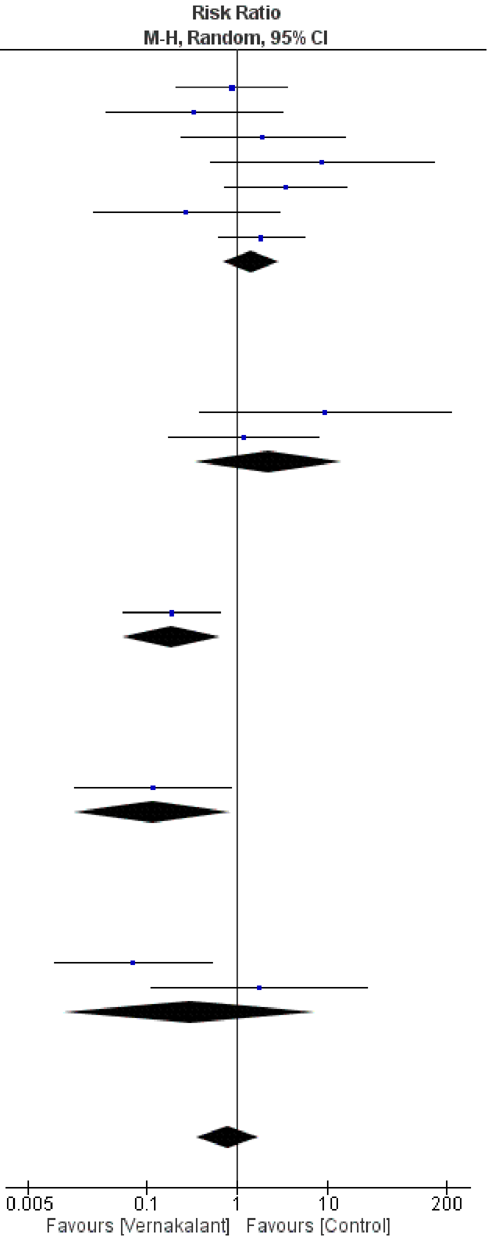

Supplementary Figure S5A. Forest plot of successful conversion to sinus rhythm in the sensitivity analysis restricted to atrial fibrillation-only studies.

Study or Subgroup	Vernakalant Events	Vernakalant Total	Control Events	Control Total	Weight	Risk Ratio M-H, Random, 95% CI
**1.1.1 Placebo**						
Beatch 2016	59	129	1	68	0.9%	31.10 [4.40, 219.60]
Beatch 2017	29	55	7	56	3.8%	4.22 [2.02, 8.81]
Roy 2004	13	36	1	20	0.9%	7.22 [1.02, 51.23]
Roy 2008	83	221	3	115	2.2%	14.40 [4.65, 44.55]
Torp-Pedersen 2011	191	445	57	160	6.8%	1.20 [0.95, 1.52]
**Subtotal (95% CI)**		**886**		**419**	**14.7%**	**6.17 [1.44, 26.37]**
Total events	375		69			
Heterogeneity: Tau² = 2.31; Chi² = 54.05, df = 4 (P < 0.00001); I² = 93%						
Test for overall effect: Z = 2.46 (P = 0.01)						
**1.1.2 Amiodarone**						
Bollmann 2013	11	15	5	15	3.5%	2.20 [1.01, 4.79]
Camm 2011	60	116	6	116	3.4%	10.00 [4.50, 22.23]
Conde 2013b	13	15	5	15	3.7%	2.60 [1.24, 5.46]
Dominijanni 2026a	100	131	327	486	7.4%	1.13 [1.01, 1.27]
**Subtotal (95% CI)**		**277**		**632**	**18.1%**	**2.76 [0.84, 9.08]**
Total events	184		343			
Heterogeneity: Tau² = 1.36; Chi² = 53.45, df = 3 (P < 0.00001); I² = 94%						
Test for overall effect: Z = 1.67 (P = 0.09)						
**1.1.3 Procainamide**						
Stiell 2025	111	178	83	172	7.1%	1.29 [1.07, 1.57]
**Subtotal (95% CI)**		**178**		**172**	**7.1%**	**1.29 [1.07, 1.57]**
Total events	111		83			
Heterogeneity: Not applicable						
Test for overall effect: Z = 2.61 (P = 0.009)						
**1.1.4 Flecainide**						
Conde 2014b	14	15	16	20	6.7%	1.17 [0.90, 1.51]
Dominijanni 2026b	101	131	105	151	7.3%	1.11 [0.96, 1.28]
Kriz 2016b	27	34	42	59	6.8%	1.12 [0.88, 1.41]
Pohjantähti-Maaroos 2017	67	100	46	100	6.7%	1.46 [1.13, 1.88]
**Subtotal (95% CI)**		**280**		**330**	**27.5%**	**1.18 [1.05, 1.33]**
Total events	209		209			
Heterogeneity: Tau² = 0.00; Chi² = 3.86, df = 3 (P = 0.28); I² = 22%						
Test for overall effect: Z = 2.69 (P = 0.007)						
**1.1.5 Propafenone**						
Conde 2014a	13	15	25	30	6.7%	1.04 [0.81, 1.34]
**Subtotal (95% CI)**		**15**		**30**	**6.7%**	**1.04 [0.81, 1.34]**
Total events	13		25			
Heterogeneity: Not applicable						
Test for overall effect: Z = 0.30 (P = 0.76)						
**1.1.6 Ibutilide**						
Kriz 2016a	27	34	73	107	6.9%	1.16 [0.94, 1.44]
Simon 2017	34	49	22	51	6.0%	1.61 [1.12, 2.32]
Vogiatzis 2017	19	36	22	42	5.7%	1.01 [0.66, 1.54]
**Subtotal (95% CI)**		**119**		**200**	**18.6%**	**1.23 [0.97, 1.56]**
Total events	80		117			
Heterogeneity: Tau² = 0.02; Chi² = 3.23, df = 2 (P = 0.20); I² = 38%						
Test for overall effect: Z = 1.74 (P = 0.08)						
**1.1.7 Electrical Cardioversion**						
Conde 2013a	27	30	30	30	7.3%	0.90 [0.79, 1.03]
**Subtotal (95% CI)**		**30**		**30**	**7.3%**	**0.90 [0.79, 1.03]**
Total events	27		30			
Heterogeneity: Not applicable						
Test for overall effect: Z = 1.52 (P = 0.13)						
**Total (95% CI)**		**1785**		**1813**	**100.0%**	**1.54 [1.26, 1.88]**
Total events	999		876			
Heterogeneity: Tau² = 0.14; Chi² = 175.75, df = 18 (P < 0.00001); I² = 90%						
Test for overall effect: Z = 4.21 (P < 0.0001)						
Test for subgroup differences: Chi² = 21.28, df = 6 (P = 0.002), I² = 71.8%						

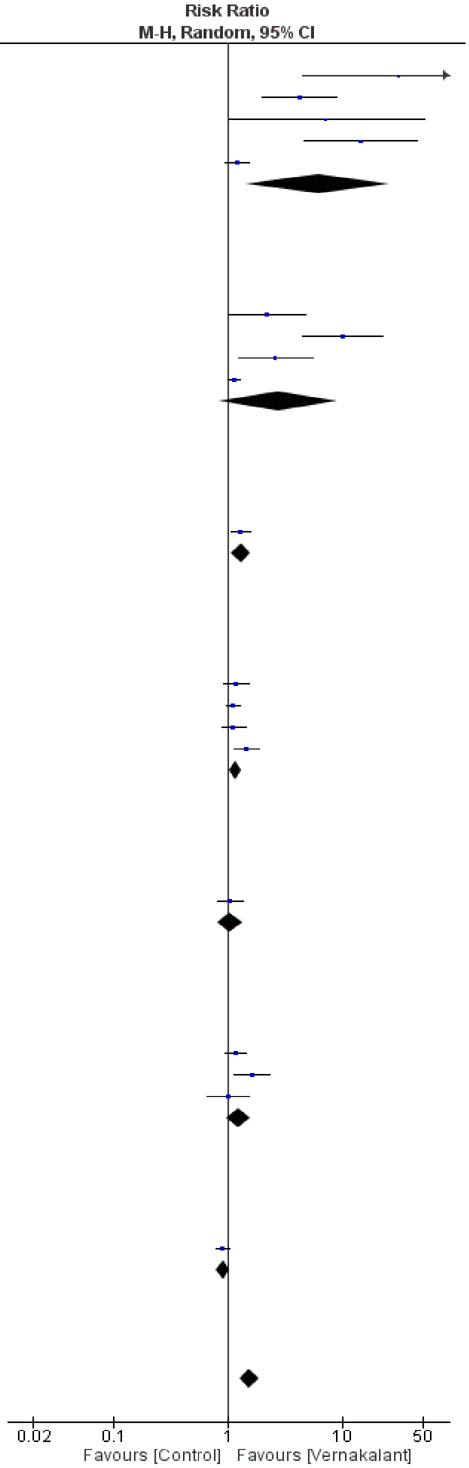

Supplementary Figure S5B. Forest plot of all-cause mortality in the sensitivity analysis restricted to atrial fibrillation-only studies.

	Vernakalant		Control			Risk Ratio
Study or Subgroup	Events	Total	Events	Total	Weight	M-H, Random, 95% CI
Beatch 2016	1	129	1	68	14.4%	0.53 [0.03, 8.30]
Beatch 2017	1	55	0	56	10.8%	3.05 [0.13, 73.38]
Camm 2011	1	116	0	116	10.8%	3.00 [0.12, 72.89]
Kriz 2016	0	68	1	107	10.8%	0.52 [0.02, 12.63]
Roy 2008	3	221	0	115	12.5%	3.66 [0.19, 70.21]
Stiell 2025	2	178	0	172	11.9%	4.83 [0.23, 99.93]
Torp-Pedersen 2011	2	548	2	184	28.7%	0.34 [0.05, 2.37]
**Total (95% CI)**		**1315**		**818**	**100.0%**	**1.12 [0.39, 3.19]**
Total events	10		4			

Heterogeneity: $Tau^2 = 0.00$; $Chi^2 = 4.34$, df = 6 (P = 0.63); $I^2 = 0\%$
Test for overall effect: Z = 0.21 (P = 0.83)

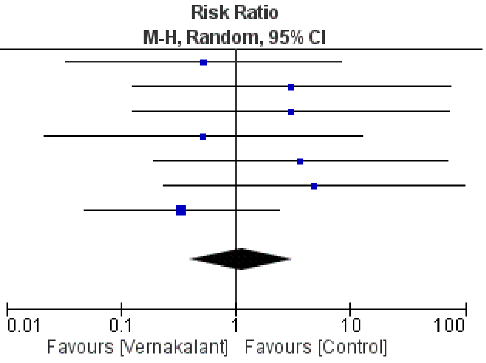

Supplementary Figure S5C. Forest plot of drug discontinuation in the sensitivity analysis restricted to atrial fibrillation-only studies.

	Vernakalant		Control			Risk Ratio
Study or Subgroup	Events	Total	Events	Total	Weight	M-H, Random, 95% CI
Beatch 2016	3	129	0	68	4.0%	3.72 [0.19, 70.90]
Camm 2011	3	116	1	116	6.5%	3.00 [0.32, 28.42]
Roy 2008	4	221	1	115	6.8%	2.08 [0.24, 18.41]
Simon 2017	0	49	1	51	3.4%	0.35 [0.01, 8.31]
Stiell 2025	4	178	15	172	20.0%	0.26 [0.09, 0.76]
Torp-Pedersen 2011	244	548	98	184	52.6%	0.84 [0.71, 0.99]
Vogiatzis 2017	1	36	3	42	6.6%	0.39 [0.04, 3.58]
**Total (95% CI)**		**1277**		**748**	**100.0%**	**0.75 [0.41, 1.37]**
Total events	259		119			

Heterogeneity: $Tau^2 = 0.18$; $Chi^2 = 8.21$, df = 6 (P = 0.22); $I^2 = 27\%$
Test for overall effect: Z = 0.94 (P = 0.35)

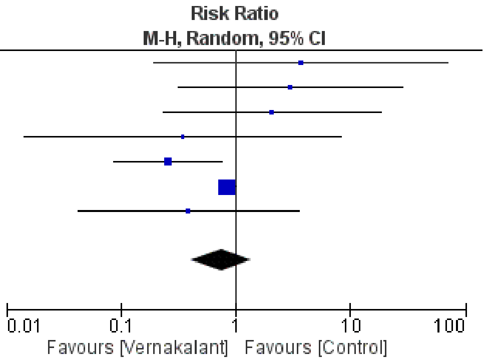

Supplementary Figure S5D. Forest plot of serious adverse events in the sensitivity analysis restricted to atrial fibrillation-only studies.

	Vernakalant		Control			Risk Ratio
Study or Subgroup	Events	Total	Events	Total	Weight	M-H, Random, 95% CI
Beatch 2016	10	129	4	68	9.1%	1.32 [0.43, 4.05]
Beatch 2017	7	55	8	56	12.5%	0.89 [0.35, 2.29]
Camm 2011	5	116	2	116	4.5%	2.50 [0.49, 12.63]
Roy 2004	1	36	4	20	2.6%	0.14 [0.02, 1.16]
Roy 2008	29	221	21	115	35.9%	0.72 [0.43, 1.20]
Torp-Pedersen 2011	46	548	18	184	35.4%	0.86 [0.51, 1.44]
**Total (95% CI)**		**1105**		**559**	**100.0%**	**0.84 [0.59, 1.19]**
Total events	98		57			

Heterogeneity: Tau² = 0.02; Chi² = 5.50, df = 5 (P = 0.36); I² = 9%
Test for overall effect: Z = 0.98 (P = 0.33)

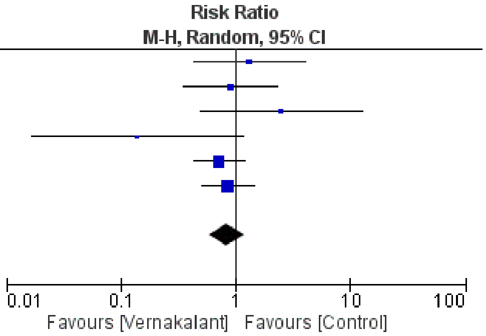

Supplementary Figure S5E. Forest plot of any ventricular tachycardia in the sensitivity analysis restricted to atrial fibrillation-only studies.

	Vernakalant		Control			Risk Ratio
Study or Subgroup	Events	Total	Events	Total	Weight	M-H, Random, 95% CI
Beatch 2016	4	129	0	68	4.6%	4.78 [0.26, 87.44]
Beatch 2017	1	55	4	56	7.9%	0.25 [0.03, 2.21]
Camm 2011	1	116	0	116	3.8%	3.00 [0.12, 72.89]
Dominijanni 2026a	1	131	0	486	3.8%	11.07 [0.45, 270.13]
Dominijanni 2026b	1	131	1	151	5.0%	1.15 [0.07, 18.25]
Kriz 2016	0	68	1	107	3.8%	0.52 [0.02, 12.63]
Roy 2004	0	36	2	20	4.3%	0.11 [0.01, 2.25]
Roy 2008	14	221	17	115	44.3%	0.43 [0.22, 0.84]
Simon 2017	0	49	7	51	4.8%	0.07 [0.00, 1.18]
Stiell 2025	1	178	5	172	8.1%	0.19 [0.02, 1.64]
Torp-Pedersen 2011	1	548	1	184	5.0%	0.34 [0.02, 5.34]
Vogiatzis 2017	0	36	3	42	4.5%	0.17 [0.01, 3.11]
**Total (95% CI)**		**1698**		**1568**	**100.0%**	**0.46 [0.24, 0.86]**
Total events	24		41			

Heterogeneity: Tau² = 0.12; Chi² = 12.07, df = 11 (P = 0.36); I² = 9%
Test for overall effect: Z = 2.42 (P = 0.02)

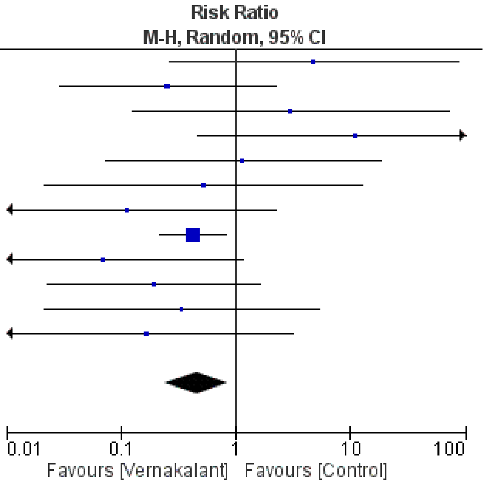

Supplementary Figure S5F. Forest plot of bradycardia in the sensitivity analysis restricted to atrial fibrillation-only studies.

Study or Subgroup	Vernakalant Events	Vernakalant Total	Control Events	Control Total	Weight	Risk Ratio M-H, Random, 95% CI
Beatch 2016	4	129	0	68	4.6%	4.78 [0.26, 87.44]
Beatch 2017	1	55	0	56	3.9%	3.05 [0.13, 73.38]
Dominijanni 2026a	2	131	24	486	14.9%	0.31 [0.07, 1.29]
Dominijanni 2026b	2	131	12	151	14.2%	0.19 [0.04, 0.84]
Kriz 2016a	1	34	0	107	3.9%	9.26 [0.39, 222.12]
Kriz 2016b	1	34	0	59	3.9%	5.14 [0.22, 122.85]
Pohjantähti-Maaroos 2017	2	100	1	100	6.6%	2.00 [0.18, 21.71]
Roy 2004	2	36	1	20	6.8%	1.11 [0.11, 11.50]
Roy 2008	1	221	0	115	3.9%	1.57 [0.06, 38.18]
Simon 2017	2	49	1	51	6.6%	2.08 [0.19, 22.23]
Stiell 2025	5	178	4	172	17.1%	1.21 [0.33, 4.42]
Torp-Pedersen 2011	9	548	2	184	13.6%	1.51 [0.33, 6.93]
**Total (95% CI)**		**1646**		**1569**	**100.0%**	**1.07 [0.55, 2.05]**
Total events	32		45			

Heterogeneity: Tau² = 0.21; Chi² = 13.15, df = 11 (P = 0.28); I² = 16%
Test for overall effect: Z = 0.19 (P = 0.85)

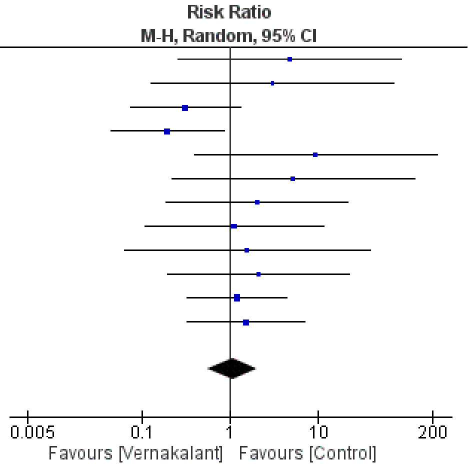

Supplementary Figure S5G. Forest plot of hypotension in the sensitivity analysis restricted to atrial fibrillation-only studies.

Study or Subgroup	Vernakalant Events	Vernakalant Total	Control Events	Control Total	Weight	Risk Ratio M-H, Random, 95% CI
Beatch 2016	5	129	3	68	13.0%	0.88 [0.22, 3.57]
Beatch 2017	1	55	3	56	8.5%	0.34 [0.04, 3.16]
Dominijanni 2026a	1	131	31	486	9.7%	0.12 [0.02, 0.87]
Dominijanni 2026b	1	131	16	151	9.5%	0.07 [0.01, 0.54]
Kriz 2016a	1	34	0	107	5.4%	9.26 [0.39, 222.12]
Kriz 2016b	1	34	1	59	6.6%	1.74 [0.11, 26.86]
Roy 2004	1	36	2	20	8.1%	0.28 [0.03, 2.88]
Roy 2008	14	221	4	115	15.1%	1.82 [0.61, 5.41]
Stiell 2025	3	178	15	172	14.2%	0.19 [0.06, 0.66]
Vogiatzis 2017	2	36	2	42	10.0%	1.17 [0.17, 7.87]
**Total (95% CI)**		**985**		**1276**	**100.0%**	**0.53 [0.22, 1.25]**
Total events	30		77			

Heterogeneity: Tau² = 0.97; Chi² = 19.89, df = 9 (P = 0.02); I² = 55%
Test for overall effect: Z = 1.46 (P = 0.15)

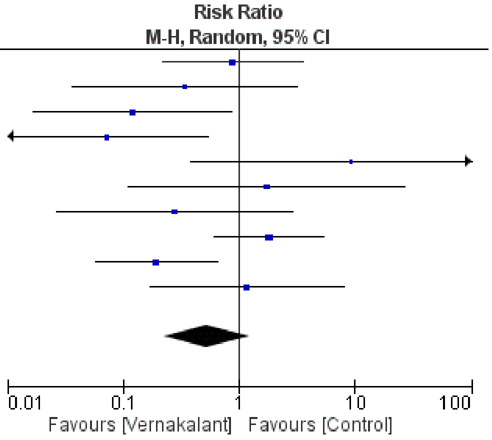

Supplementary Table S1. Quality assessment of included cohort studies using the Newcastle–Ottawa Scale (NOS).

Study ID	Selection				Comparability	Outcome			Total
	Representativeness of the Exposed Cohort	Selection of the Non-Exposed Cohort	Ascertainment of Exposure (1 points)	Demonstration That Outcome of Interest Was Not Present at Start of Study	Comparability of Cohorts on the Basis of the Design or analysis	Assessment of Outcome	Was Follow-Up Long Enough for Outcomes to Occur	Adequacy of Follow Up of Cohorts	
**Lindmayr 2022**	☆	☆	☆	☆		☆	☆	☆	☆☆☆☆☆☆☆
**Kriz 2016**	☆	☆	☆	☆	☆	☆	☆	☆	☆☆☆☆☆☆☆☆
**Pohjantähti-Maaroos 2017**	☆	☆	☆	☆	☆	☆	☆	☆	☆☆☆☆☆☆☆☆
**Conde 2013a**		☆	☆	☆		☆	☆	☆	☆☆☆☆☆☆
**Conde 2013b**	☆	☆	☆	☆			☆	☆	☆☆☆☆☆☆
**Conde 2014**	☆	☆	☆	☆		☆	☆	☆	☆☆☆☆☆☆☆
**Dominijanni 2026**	☆	☆	☆	☆	☆	☆	☆	☆	☆☆☆☆☆☆☆☆

Supplementary Table S2. Summary of GRADE certainty of evidence for all evaluated outcomes.

Outcome	Sample Size	Effect Estimate	Quality Assessment	Inconsistency	Indirectness	Imprecision	Publication Bias	Overall Quality
**Serious adverse events**	2,144	0.87 (0.67–1.14)	Not serious	Not serious	Not serious	Not serious	N/A	Moderate
**All-Cause Mortality – Clinical Trials**	2223 (7)	1.35 (0.47, 3.84)	Not serious	Not Serious	Not Serious	Serious	Serious	Low
**All-Cause Mortality – Observational Studies**	175 (1)	0.52 (0.02, 12.63)	Not serious	N/A	Not Serious	Serious	Serious	Very low
**Any Ventricular Tachycardia – Clinical Trials**	2618 (11)	0.61 (0.33, 1.12)	Not serious	Not Serious	Not Serious	Serious	Not serious	Moderate
**Any Ventricular Tachycardia – Observational Studies**	1074 (3)	1.79 (0.31, 10.25)	Not serious	Serious	Not Serious	Serious	Not serious	Very Low
**Bradycardia – Clinical Trails**	2362 (10)	2.21 (1.16, 4.20)	Not serious	Not serious	Not Serious	Serious	Serious	Low
**Bradycardia – Observational**	1649 (6)	0.57 (0.21, 1.58)	Serious	Serious	Not Serious	Serious	Serious	Very Low

**Hypotension – Clinical Trials**	1608 (9)	1.00 (0.45, 2.18)	Serious	Serious	Not Serious	Serious4	Not serious	Low
**Hypotension – Observational Studies**	1133 (4)	0.47 (0.06, 3.80)	Serious	Serious	Not Serious	Serious	Not serious	Very Low
**Successful Conversion to Sinus Rhythm – Clinical Trials**	2572(13)	3.19 (1.97–5.15)	Serious	Serious	Not Serious	Not serious	Serious	Low
**Successful Conversion to Sinus Rhythm – Observational Studies**	1819 (10)	1.11 (1.01, 1.22)	Serious	Not serious	Not Serious	Not Serious	Serious	Very Low
**Drug Discontinuation – Clinical Trials**	2505 (10)	0.98 (0.51, 1.88)	Serious	Serious	Not Serious	Serious	Not serious	Low

Supplementary Table S3. Egger's regression test for small-study effects across study outcomes.

Outcome	P-value (2-tailed)	95% CI (2-tailed)
**Successful Conversion to Sinus Rhythm**	0.02	0.40 to 3.78
**SAEs**	0.84	−2.19 to 1.83
**Drug Discontinuation**	0.18	−0.41 to 1.85
**Bradycardia**	<0.01	0.92 to 3.89
**Hypotension**	0.72	−2.61 to 3.67
**Any Ventricular Tachycardia**	0.62	−1.62 to 1.01
**Mortality**	0.03	0.40 to 5.96

Search Strategy

PubMed: (Vernakalant[title/abstract] OR Brinavess[title/abstract] OR RSD1235[title/abstract]) AND ("Atrial fibrillation"[title/abstract] OR Afib[title/abstract] OR A-fib[title/abstract] OR "Atrial-fibrillation"[title/abstract] OR "Atrial-fib"[title/abstract] OR "Atrial fib*"[title/abstract] OR fibrillation[title/abstract] OR flutter[title/abstract])

Embase: ('vernakalant'/exp OR vernakalant:ti,ab OR brinavess:ti,ab OR RSD1235:ti,ab) AND ('atrial fibrillation'/exp OR 'atrial flutter'/exp OR 'atrial fibrillation':ti,ab OR AFib:ti,ab OR 'A-fib':ti,ab OR 'atrial-fibrillation':ti,ab OR 'atrial-fib':ti,ab OR 'atrial fib*':ti,ab OR fibrillation:ti,ab OR flutter:ti,ab)

CENTRAL: (vernakalant OR Brinavess OR RSD1235):ti,ab,kw AND ("atrial fibrillation" OR AFib OR "A-fib" OR "atrial-fibrillation" OR "atrial-fib" OR (atrial NEXT fib*) OR fibrillation OR flutter):ti,ab,kw
